# Dynamics and control of the ERK signaling pathway: Sensitivity, bistability, and oscillations

**DOI:** 10.1371/journal.pone.0195513

**Published:** 2018-04-09

**Authors:** Yaman Arkun, Mohammadreza Yasemi

**Affiliations:** Department of Chemical and Biological Engineering, Koc University, Rumeli Feneri Yolu, Sariyer, Istanbul, Turkey; Tata Institute of Fundamental Research, INDIA

## Abstract

Cell signaling is the process by which extracellular information is transmitted into the cell to perform useful biological functions. The ERK (extracellular-signal-regulated kinase) signaling controls several cellular processes such as cell growth, proliferation, differentiation and apoptosis. The ERK signaling pathway considered in this work starts with an extracellular stimulus and ends with activated (double phosphorylated) ERK which gets translocated into the nucleus. We model and analyze this complex pathway by decomposing it into three functional subsystems. The first subsystem spans the initial part of the pathway from the extracellular growth factor to the formation of the SOS complex, ShC-Grb2-SOS. The second subsystem includes the activation of Ras which is mediated by the SOS complex. This is followed by the MAPK subsystem (or the Raf-MEK-ERK pathway) which produces the double phosphorylated ERK upon being activated by Ras. Although separate models exist in the literature at the subsystems level, a comprehensive model for the complete system including the important regulatory feedback loops is missing. Our dynamic model combines the existing subsystem models and studies their steady-state and dynamic interactions under feedback. We establish conditions under which bistability and oscillations exist for this important pathway. In particular, we show how the negative and positive feedback loops affect the dynamic characteristics that determine the cellular outcome.

## Introduction

Signal transduction pathways consist of signaling proteins that communicate through complex molecular mechanisms. The network of biomolecular interactions and feedback loops are usually arranged in a hierarchical fashion in space and time to transform the information content of extracellular signals to the DNA in the nucleus which in turn converts this information into several useful cellular functions [1]. The signal transduction pathway under study controls the ERK (extracellular signal-regulated kinase) signaling which plays a key role in numerous cellular processes such as proliferation, DNA synthesis, differentiation and apoptosis [2],[3]. Signaling faults due to mutations and failures in the regulatory mechanisms of the ERK signaling pathway are known to result in several types of cancer [4]. Therefore, the ERK pathway has been a common target for the treatment of cancer [2] [3] [5] [6] [7].

**Fig 1:**
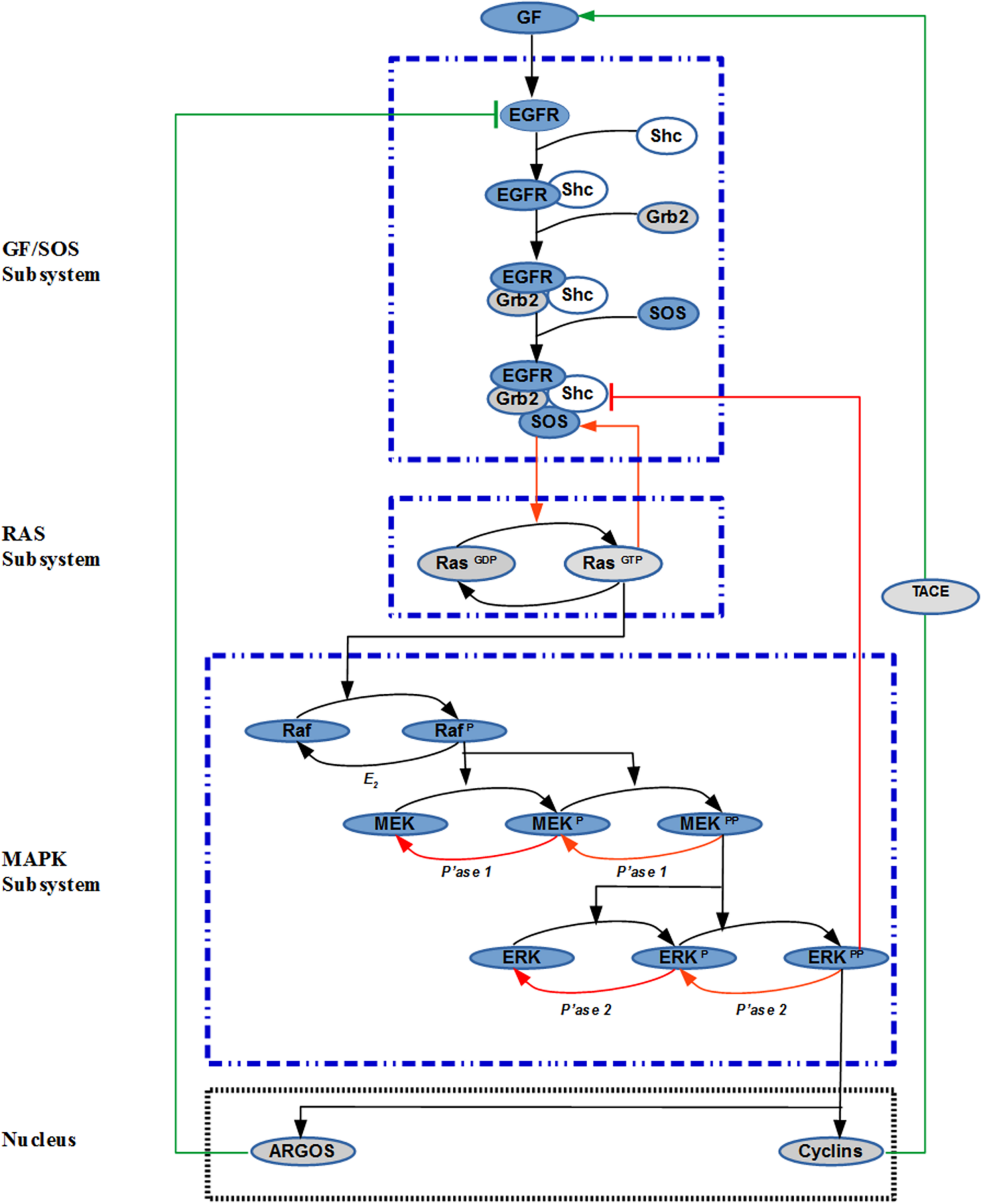
The ERK signaling pathway. Different subsystems and feedback loops are hierarchically organized to process the extracellular signal introduced by Growth Factor (GF). Red arrows indicate internal positive feedback loops. Green arrows represent the external feedback loops. Blue dashed lines and black dashed lines indicate the subsystems and the nucleus, respectively.

In cells from yeast to mammals, receptor tyrosine kinases (RTKs) are known to exploit a highly conserved signal-transduction pathway, carrying the signal triggered by binding of growth factors to activate ERK [8] [9]. The ERK signaling pathway is one of the most important and intensively studied signaling pathways [5] [10]. It is comprised of three subsystems as shown in Fig 1. Activation of EGFR (epidermal growth factor receptor) by binding of its specific ligands, namely epidermal growth factor (EGF) and transforming growth factor α (TGF α), starts the ERK signaling pathway. Upon ligand binding, two subunits of EGFR are dimerized, leading to increase in the enzymatic activity of its cytoplasmic tyrosine kinase domain [11]. Adapter protein ShC-Grb2 binds to the phosphorylated RTK and recruits SOS leading to the formation of the ShC-Grb2-SOS complex [12] [13]. Membrane-bound protein Ras, which is a small GTP binding protein, interacts with the ShC-Grb2-SOS complex, and it is transformed to its active conformation by exchanging GDP for GTP. Active Ras serves as an important molecular switch which starts the sequential phosphorylation of the MAPK pathway that consists of the Raf-MEK-ERK signaling cascade [14] [15].

Activated ERK, when translocated to the nucleus, phosphorylates certain nuclear transcription factors (e.g. Myc), that govern the cellular responses [16]. Phosphorylated transcription factors stimulate transcription of genes which are responsible for encoding different proteins, including the ones required for cell cycle progression (e.g. Cyclin D) [17].

The information necessary to perform different cellular functions is encoded in the dynamics of the ERK signal. Duration, amplitude, stability and frequency of this signal have to be regulated in order to control the biological outcomes [18] [19]. For example, Brightman et al. [20] has observed that the EGF-induced pathway demonstrates the transient ERK dynamics, while the same pathway exhibits sustained ERK activation when induced by NGF (nerve growth factor), each ending up in a different cell fate in PC12 cells [1][20][21]. This observed different dynamics of ERK has been attributed to the negative feedback inhibition of the MAPK subsystem [20].

A central question that this paper tries to address is: how can the pathway dynamics be modulated to generate a diverse array of ERK dynamics that will selectively affect the cellular functions? In order to answer this fundamental question, we study the effect of different feedback loops (FBLs) as shown in Fig 1. Those loops introduce complex dynamic interactions among the different subsystems which are difficult to decipher unless a comprehensive dynamic model is available for the whole pathway.

For engineering systems, the feedback loops are designed by humans; for biological systems evolution has designed them to fulfill certain important biological functions. We categorize the feedback loops into two classes. Type 1: these are the kinetically built-in *internal feedback loops* which reside within the pathway (see Fig 1). Type 2: these are the *external feedback loops* that are not embedded within the pathway. Instead they affect the pathway through molecular species (e.g. TACE and ARGOS in Fig 1) that are outside the main signaling pathway. TACE, also known as ADAM17, is synthesized in the endoplasmic reticulum and matures in a late Golgi compartment [22]. Similarly, ARGOS is produced in the nucleus. Unlike the internal feedback loops, both TACE and ARGOS act externally on the cell membrane. External and internal feedback loops regulate the ERK dynamics (duration, magnitude, oscillations etc.) by coordinating their actions with each other. Below an explanation is provided for the origin of each type of feedback loop.

### Internal feedback loops

There are four internal feedback loops, three of which are positive and one is negative.

The first internal feedback loop IFBL1 is the positive feedback loop within the Ras subsystem. Das et al. [14] proposed a mechanism for this positive feedback loop in lymphoid cells which includes (1) catalytic activation Ras by Shc-Grb2-SOS while RasGTP (already activated form of Ras) is bound to its allosteric site, and (2) deactivation of RasGTP by RasGAP (Ras GTPase activating protein). This positive loop creates a bistable response for RasGTP and acts as a gate switch for the propagation of the signal from the growth factor to ERK downstream in the pathway.

The second set of internal positive feedback loops IFBL2 and IFBL3 are due to the dual phosphorylation-dephosphorylation cascades embedded within the MAPK pathway. These reactions introduce positive feedback and can lead to ultrasensitive switch-like responses with bistability and hysteresis [23][24]. It is well-known that the two-site MAPK phosphorylation and dephosphorylation cycle with a distributive kinetic mechanism for the kinase and phosphatase possesses the necessary properties to exhibit bistable response [25] [26]. MEK and MEKP compete for the same kinase for phosphorylation; MEKPP and MEKP compete for the same phosphatase for dephosphorylation. Through this competition, MEK inhibits the production of MEKPP, and MEKPP inhibits the production of MEK. This double inhibition results in the positive feedback loop IFBL2 which leads to bistability under the right set of operating conditions or parameter values. The same argument can be made for the last stage of phosphorylation and dephosphorylation reactions of ERK and positive feedback loop IFBL3.The last internal feedback loop IFBL4 acts as a negative loop between the MAPK and GF/SOS subsystems. Active ERK catalyzes SOS phosphorylation which in turn leads to dissociation of the SOS complex [27] [28]; thus forming a negative loop around the SOS complex.

### External feedback loops

The first external feedback loop EFBL1 is the positive autocrine feedback loop that acts on the cell membrane. In autocrine loops, ligands secreted by cells (GFs) bind and stimulate the receptors of the same cells (EGFRs) [29] [30]. In such loops, proteases, (e.g. TACE), known as *sheddases*, cleave the secreted ligands from the outer membrane of the cell and make it available for signaling and ERK activation. At the same time, TACE production is positively induced by ERK which is activated by the cleaved ligands. Since the pathway and ERK are stimulated by the ligands they release, a positive feedback loop is established [29].

TACE (tumor necrosis factor a-converting enzyme) also known as ADAM17 sheds a large number of EGFR ligands and it is known to be activated by ERK [31][32]. While the exact mechanism of activation remains unclear, it has been reported that active ERK phosphorylates TACE at Thr735 leading to TACE maturation and trafficking to the cell surface. TACE autocrine loop has to be tightly regulated since uncontrolled release of ligands can result in autoimmune diseases, chronic inflammation and cancer progression [33].

The second external feedback loop EFBL2 is the negative feedback loop acting between the nucleus and the extracellular domain. ARGOS is a secreted protein which interacts directly with the EGFR leading to inhibition of ERK signaling. Transcriptional programs induced by ERK lead to production of ARGOS in the nucleus [29] [34]. In turn ARGOS acts as a scavenger of the EGF-like ligands [35] by either binding to EGFR and inhibiting its binding with GFs or by directly sequestrating ligands right after their secretion [35] [36]. Both mechanisms downregulate the EGFR signaling.

Next we present the methods used to model and analyze the ERK signaling pathway including the feedback loops described above.

## Methods

Use of systems biology tools and computational approaches in molecular biology have made the analytical investigation of intracellular dynamics possible. Systems biology adopts a holistic approach in general and turns the abstract biological descriptions into mathematical models and computational formalisms. Knowing the individual proteins involved in a complex pathway is not enough to describe how a particular signal transduction pathway functions. In systems approach, the system is analyzed as a whole by paying attention to the interactions among its detached subsystems [37].

Mathematical models are useful in many ways. First, they can be used to discriminate among the alternative causal mechanisms. Based on the model predictions, new hypotheses can be postulated and experiments can be designed to validate these hypotheses. Investigations being carried out based on mathematical models which mimic the intracellular networks, have achieved much attention in recent years [37][38]. Well-documented recurring motifs like ultra-sensitivity, bistability and oscillations are a few valuable gains of mathematical investigation of biological systems [20][39].

Informative models explaining different parts of the ERK signaling pathway were constructed by several authors [12][13][40][41]. Usually each model is tailored in a way that fulfills the demands upon which it is built. In this paper, we distinguish between the model for the signaling pathway and the model for the feedback controller. The complete model consists of the integration of these two models under closed-loop control. This approach helps us to better structure and explain the interaction of the feedback loops with the signaling pathway.

We have developed a quantitative mechanistic dynamic model including mass conservation and mass action kinetics for the complete system presented in Fig 1. This model is constructed by combining some of the current models available for the subsystems shown in Fig 1. The developed model was analyzed to understand the steady-state and dynamic properties of the ERK signaling under feedback control. In particular, bifurcation analysis proved to be a very useful technique in obtaining enlightening results that explain the behavior of this complex dynamical system.

Next we describe the models used for each subsystem of our signaling pathway without the feedback loops. This is followed by the description of how these models were integrated together and how the feedback loops were modeled and added.

### Modeling of the GF-SOS subsystem

In 1999 Kholodenko et al. [12] derived a kinetic model of EGFR signaling pathway to describe the complex cellular responses to EGF. We have adopted this model for our GF-SOS subsystem. The model includes reactions and species starting with the extracellular stimulus and ends with the formation of the SOS complex, ShC-Grb2-SOS (see Fig 1) which will be abbreviated as SOS_complex_. The model has received much attention because it has successfully incorporated EGFR and its adapter proteins. The model and the values for its parameters are given in S1 Text.

### Modeling of the Ras subsystem

This model represents the activation of Ras and is modeled after Das et al. [14]. The allosteric reactions produce SOS_complex_ -RasGDP and SOS_complex_ -RasGTP each of which catalyzes the activation of RasGDP to RasGTP by different extents. Since SOS_complex_ -RasGTP is more than 75 times active than SOS_complex_−RasGDP, it is the dominant catalyst of Ras activation. For modeling activation/deactivation reactions of the RasGDP/RasGTP, Michaelis-Menten kinetics was employed, while allosteric reactions were modelled by mass action kinetics (see S2 Text).

### Modeling of the MAPK subsystem

The model represents the MAPK cascade. Activated Ras *(*Ras-GTP*)*, is the input to the model and consequently three kinases, namely, Raf, MEK and ERK become activated. Each activated kinase is the enzyme for the activation of the next step. MEK and ERK are fully activated when they are double phosphorylated. Specific phosphatase of each active kinase dephosphorylates it and makes it inactive. This model (see S3 Text) is borrowed from [24].

### Interfacing the subsystem models

The subsystem models that we have borrowed from the literature had to be combined in order to form a single model for the entire ERK signaling pathway. This must be done systematically to make sure that no critical information is lost. At the same time, the resulting model should not be unduly complicated, allowing transparent analysis. Several steps were taken to tackle these issues. First, the units of all the parameters involved in the individual models given in different units were converted to a single set of units for the complete model. We chose seconds for time and nano-molar for concentration. The parameters of the GF-SOS subsystem model and the MAPK subsystem model were fixed at their literature values given in [12] and [24], respectively. However some of the parameters of the RAS model given in [14] had to be tuned for the reasons explained next.

The input to the RAS subsystem is the *SOS*_*complex*_ from the upstream GF-SOS subsystem. The output of the RAS subsystem model, RasGTP, triggers the ERK signaling by activating the downstream MAPK subsystem. It is well-known that RasGTP exhibits a bistable switching response which propagates the signal to downstream MAPK pathway. Therefore, when connecting the RAS model to the GF-SOS subsystem, some of its parameters had to be adjusted to assure that RasGTP maintained its desired bistable response with hysteresis for the range of the *SOS*_*complex*_ concentrations provided by the upstream GF-SOS subsystem model. After performing a sensitivity analysis (see S2 Text), we chose to tune the rate constant kcat3 and the Michealis constant K5m which affect the allosteric activation and deactivation of RasGTP, the two most important reactions that control the strength of positive feedback. After these parameters were carefully adjusted, the RasGTP response maintained a bistable regime with a sharp switch between its two stable steady-states for the given concentration values in the GF-SOS and RAS subsytems.

### Modeling of the internal positive feedback loops IFBL1, IFBL2 and IFBL3

It is usually challenging to isolate the internal positive feedback loops as they emerge from complex kinetics which camouflage their inputs and outputs. The first internal feedback loop IFBL1 is due to the catalytic activation of Ras at the allosteric site of Shc-Grb2-SOS when the site is occupied by the active form of Ras, RasGTP.

The feedback is modeled by the mass balances of the species forming the positive feedback loop and is given by:

$$\begin{array}{l} \frac{d\left[RasGTP\right]}{dt}=-kfi2*\left[SOS_{complex}\right]*\left[RasGTP\right]+kbi2*\left[SOS-RasGTP\right]+kcati3\\ \;*\left[RasGDP\right]*\frac{\left[SOS-RasGTP\right]}{Ki3m+\left[RasGDP\right]}+kcati4*\left[RasGDP\right]*\frac{\left[SOS-RasGDP\right]}{Ki4m+\left[RasGDP\right]}\\ \;-kcati5*\left[GAPS\right]*\frac{\left[RasGTP\right]}{Ki5m+\left[RasGTP\right]}-a1*\left[Raf\right]*\left[RasGTP\right]+\left(d1+k1\right)\\ \;*\left[Raf-RasGTP\right] \end{array}$$ (1)

$$\frac{d[SOS-RasGTP]}{dt}=kfi2*[SOS_{complex}]*[RasGTP]-kbi2*[SOS-RasGTP]$$ (2)

The term with the parameter *kcati3* represents the reaction when the allosteric pocket is occupied by RasGTP. The rest of the terms include the reactions outside the loop (see the Ras subsystem model in S2 Text).

Two additional internal positive feedback loops, IFBL2 and IFBL3, are formed from phosphorylation and dephosphorylation of MEK and ERK in stage 1 and stage 2 of the MAPK cascade, respectively. The mechanisms that lead to positive feedback are identical for each loop.

It has been theoretically shown that positive feedback emerges from the dual phosphorylation–dephosphorylation cycle of MAPK and renders the pathway bistable [25]. This has also found experimental support for Xenopus oocytes [42]. In our MAPK model, only the first stage (i.e. MEK) exhibits bistability; therefore, the corresponding loop IFBL2 provides the switch while IFBL3’s effect is to scale the response for ERK (see Results).

### Modeling of the internal negative feedback loop IFBL4

This is a negative feedback loop where ERK^PP^ regulates itself by inhibiting the SOS complex. ERK^PP^ catalyzes SOS phosphorylation which in turn leads to dissociation of the SOS complex [27][43]. Therefore, we include the rate of disassociation in the GF-SOS subsystem model by adding it to the differential mass balance of SOS_complex_ (see the last term):

$$\begin{array}{l} \frac{d\left[SOS_{complex}\right]}{dt}=-kf23*\left[SOS_{complex}\right]+kb23*\left[Shc-P\right]*\left[Grb2-SOS\right]+kf22\\ \;*\left[Shc-Grb2\right]*\left[SOS\right]-kb22*\left[SOS_{complex}\right]+kf20\\ \;*\left[\left(EGF-EGFR\right)2-Shc-Grb2-SOS\right]-kb20*\left[SOS_{complex}\right]\\ \;*\left[\left(EGF-EGFR\right)2-P\right]-kfi1*\left[SOS_{complex}\right]*\left[RasGDP\right]+kbi1\\ \;*\left[SOS-RasGDP\right]-kfi2*\left[SOS_{complex}\right]*RasGTP+kbi2*\left[SOS-RasGTP\right]\\ \;-k_{nfb}*\left[SOS_{complex}\right]*\frac{\left[ERK^{PP}\right]}{Km_{nfb}+\left[SOS_{complex}\right]} \end{array}$$ (3)

The negative feedback term is given by:

$$-k_{\mathrm{nfb}}*[\mathrm{SO}S_{\mathrm{complex}}]*\frac{\left[\mathrm{ER}K^{\mathrm{PP}}\right]}{Km_{\mathrm{nfb}}+[\mathrm{SO}S_{\mathrm{complex}}]}$$ (4)

where we employed *Michaelis-Menten* kinetics. Parameters *k*_*nfb*_ *and Km*_*nfb*_ reflect the feedback strength.

### Modeling of the external feedback loops EFBL 1 and EFBL 2

**Fig 2:**
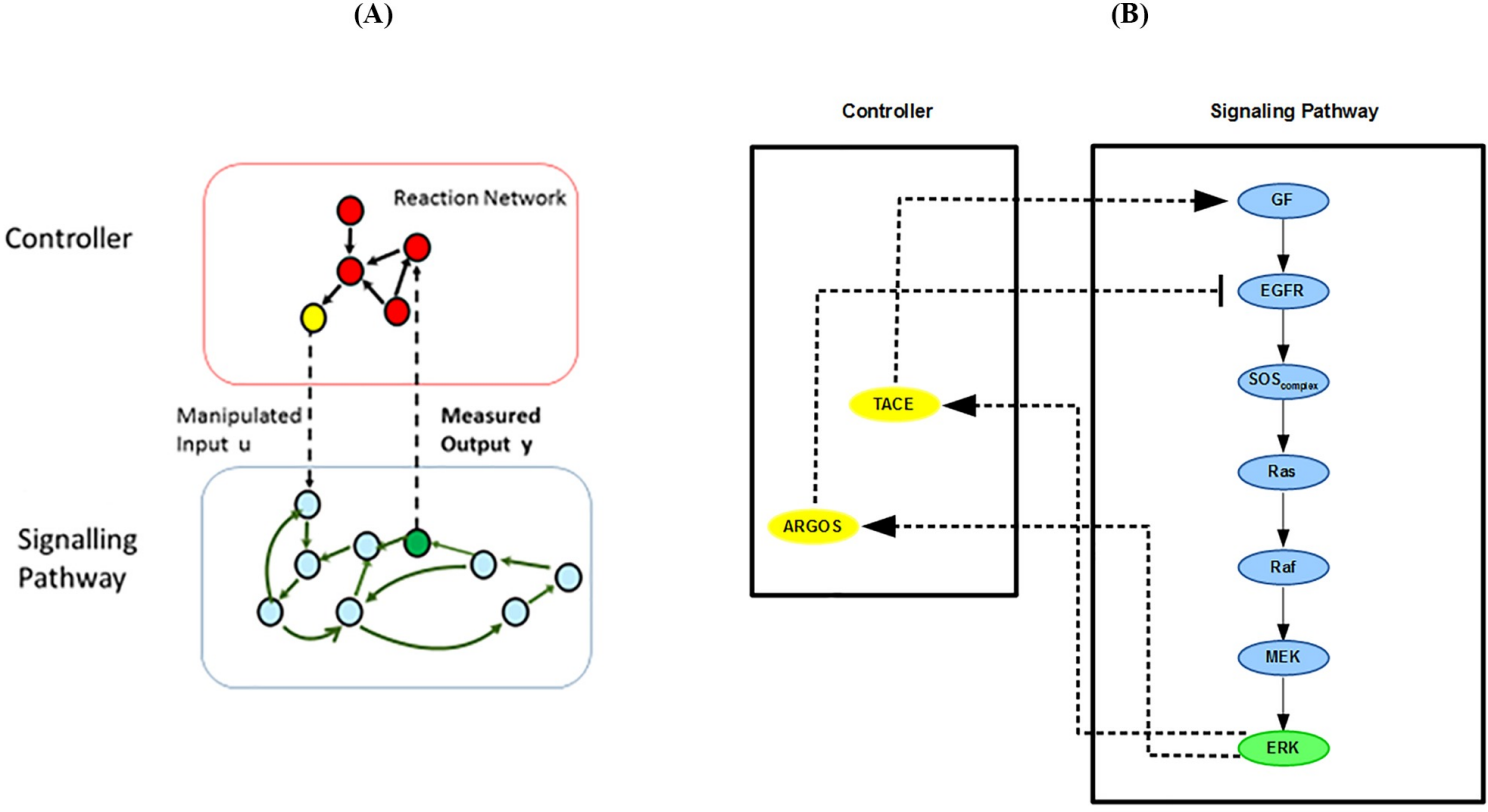
The closed-loop system. (A) Controller (reaction network), signaling pathway, measured output (green species) and manipulated input (yellow species). (B) The external feedback controller interacting with the pathway.

External feedback loops are usually more transparent than the internal loops as it is easier to identify their outputs and inputs. External loops conform the general structure of a regulatory feedback controller shown in Fig 2. This feedback structure consists of controlled output(s), manipulated input(s) and a control mechanism that defines the relationship between these two sets of variables. For a signaling pathway, the control mechanism can be seen as a separate reaction network that manipulates the input(s) to control the major output variable(s) of the pathway.

In this paper, we model the signaling pathway and its (external) feedback controller separately. This helps us to better identify and explain the interaction of the feedback loops with the signaling pathway. The components of the feedback system in Fig 2 can be mathematically expressed as:

#### Signaling pathway model

$$\frac{dx}{dt}=f_{x}(x,u),$$ (5)

y=g(x)

#### Controller model

$$\frac{dz}{dt}=f_{z}(z,y),$$ (6)

u=c(z,y)

where y is the vector of outputs, u is the vector of inputs; x and z are the states of the pathway and controller models, respectively. Both pathway and its controller are nonlinear dynamical systems in general. The signaling pathway model (5) consists of the nonlinear ODEs that model the GF-SOS, the Ras, and the MAPK subsystems (see S5 Text). The model for the external feedback loops is represented by Eq 6 and explained next.

### Modeling of the external positive autocrine loop EFBL 1

EFBL 1 is the autocrine positive feedback loop which adjusts the input GF, through TACE, to positively influence the output ERK’s activity (see Fig 2B). Production of TACE is driven by ERK and is modeled by a Hill function [30][44]:

$$\frac{d[TACE]}{dt}=gpT*\frac{{\left[ERK^{PP}\right]}^{n}}{KmT^{n}+{\left[ERK^{PP}\right]}^{n}}-dT*[TACE]$$ (7)

This differential equation is part of the controller model (6). The feedback strength is denoted by gpT.

TACE applies its positive feedback effect on ERK by increasing the released amount of active growth factor GF. Therefore, the model becomes:

$$GF_{\mathrm{available}}=GF_{\mathrm{tot}}+\mathrm{grT}*[\mathrm{TACE}]-\sum GF_{\mathrm{complex}}$$ (8)

The second term on the right-hand side of Eq 8 gives the amount of cleaved GF. GF_tot_ is the total GF without any autocrine effect; grT*[TACE] is the amount added through autocrine feedback, and the last term is the amount of unavailable GF complexes formed with other species. Eq 8 is included in the signaling pathway model.

The ligand generation grT*[TACE] is controlled by TACE activity and has zero order with respect to the amount of cell-associated ligand precursor which is in excess. This has been found to be true in some of the experimental EGFR systems and used by Pribyl et al. [44]. Since the functional form is not available, the simplest linear dependence was assumed as done in [44] where the parameter grT is called the linear gain in ligand production. Since its exact value is not available, we have performed a sensitivity analysis and shown how robust the system behavior is with respect to the parameter values (see Results).

In the above autocrine model we have assumed that the diffusion effects are not significant. Binding of autocrine ligands to their surface receptors is a two-step process consisting of its molecular transport (usually diffusion) to the receptor followed by the binding reaction [45]. Thus, the overall resistance to binding is the sum of diffusion and reaction resistances:

$$\frac{1}{k_{f}}=\frac{1}{k_{+}}+\frac{1}{{Rk}_{on}}$$ (9)

where *k*_*f*_ is the overall or observable binding rate constant; *k*_*on*_ is the intrinsic binding rate constant; *k*_+_ = 4*πDa* is the diffusion rate constant; *R* is the number of free surface receptors; *a* is the cell radius and *D* is the diffusivity. The key criterion to decide whether the process is diffusion limited or not is determined by the ratio of the ligand binding rate to diffusive rate constant i.e.

$$\rho_{cell}=\frac{Rk_{on}}{4\pi Da}$$ (10)

If *ρ*_*cell*_ ≪ 1 or *Rk*_*on*_ ≪ 4*πDa*, the ligand binding is not diffusion limited but controlled by reaction. In this paper, we assume that the conditions are such that we operate in a regime without diffusion limitations. This assumption depends very much on the individual application. For example, considering EGF binding to its normal human fibroblast [46] with *R* ~ 10^4^ receptors/cell, *ρ*_*cell*_~0.005 ≪ 1 and diffusion is not the limiting step for EGF binding [45]. However, for A431 (epidermoid carcinoma cell line) with *R* > 10^6^, *ρ*_*cell*_ > 0.5 which makes diffusion significant.

Under the assumed reaction-limited conditions, the chemical reaction is slow compared to diffusion, and the system acts as if it were well mixed with a uniform ligand concentration [47]. Therefore, our model does not include any spatial gradients which would be the case under diffusion control.

### Modeling of the external negative feedback loop EFBL 2

EFBL 2 is the inhibitory feedback loop which adjusts the input ARGOS to suppress ERK’s activity (see Fig 2B). Transcriptional programs induced by ERK lead to the production of the inhibitor ARGOS in the nucleus. Similar to the case of TACE, production of ARGOS depends on ERK and is also modeled by a Hill function [30][44]:

$$\frac{d[ARGOS]}{dt}=gpA*\frac{{\left[ERK^{PP}\right]}^{n}}{KmA^{n}+{\left[ERK^{PP}\right]}^{n}}-dA*[ARGOS]$$ (11)

This differential equation is part of the controller model (6).

Produced ARGOS binds to the available EGFR and inhibits it by forming [ARGOS – EGFR] complex [35][36]. We have modeled this negative feedback mechanism by

$$\frac{d[\mathrm{ARGOS}-\mathrm{EGFR}]}{\mathrm{dt}}=\mathrm{kAE}*[\mathrm{EGF}R_{\mathrm{available}}]*[\mathrm{ARGOS}]-\mathrm{dAE}*[\mathrm{ARGOS}-\mathrm{EGFR}]$$ (12)

Then the total amount of available EGFR is reduced accordingly:

$$EGFR_{available}=EGFR_{tot}-grA*[ARGOS-EGFR]-\sum \mathrm{EGF}R_{\mathrm{complex}}$$ (13)

Both Eq 11 and Eq 12 are included in the signaling pathway model.

## Results and discussion

The developed dynamic model can be simulated for different values of inputs e.g growth factor and model parameters. Signaling subsystems can be simulated either individually or as a whole to assess the effect of interactions among them. While internal positive feedback loops are integral part of the pathway model, the external feedback loops can be turned on and off to assess their effect. For all the scenarios considered, bifurcation analysis is performed to identify the stable, unstable, switching and oscillatory regimes of the ERK dynamics. Finally, dynamic simulations can be undertaken to demonstrate how ERK and other signaling molecules respond to extracellular changes and to the feedback loops. In the plots, bifurcation and dynamic responses are given for the most significant species that explain the significant behavior of each subsystem. These variables are SOS_complex_, RasGTP, MEK^PP^ and ERK^PP^.

### Major findings of the pathway analysis

We first analyze how the extracellular signal introduced by the growth factor is modified as it propagates through the different subsystems when external loops are off (i.e. open-loop simulation).

#### Internal positive feedback loops create ultrasensitive, bistable, switching responses

**Fig 3:**
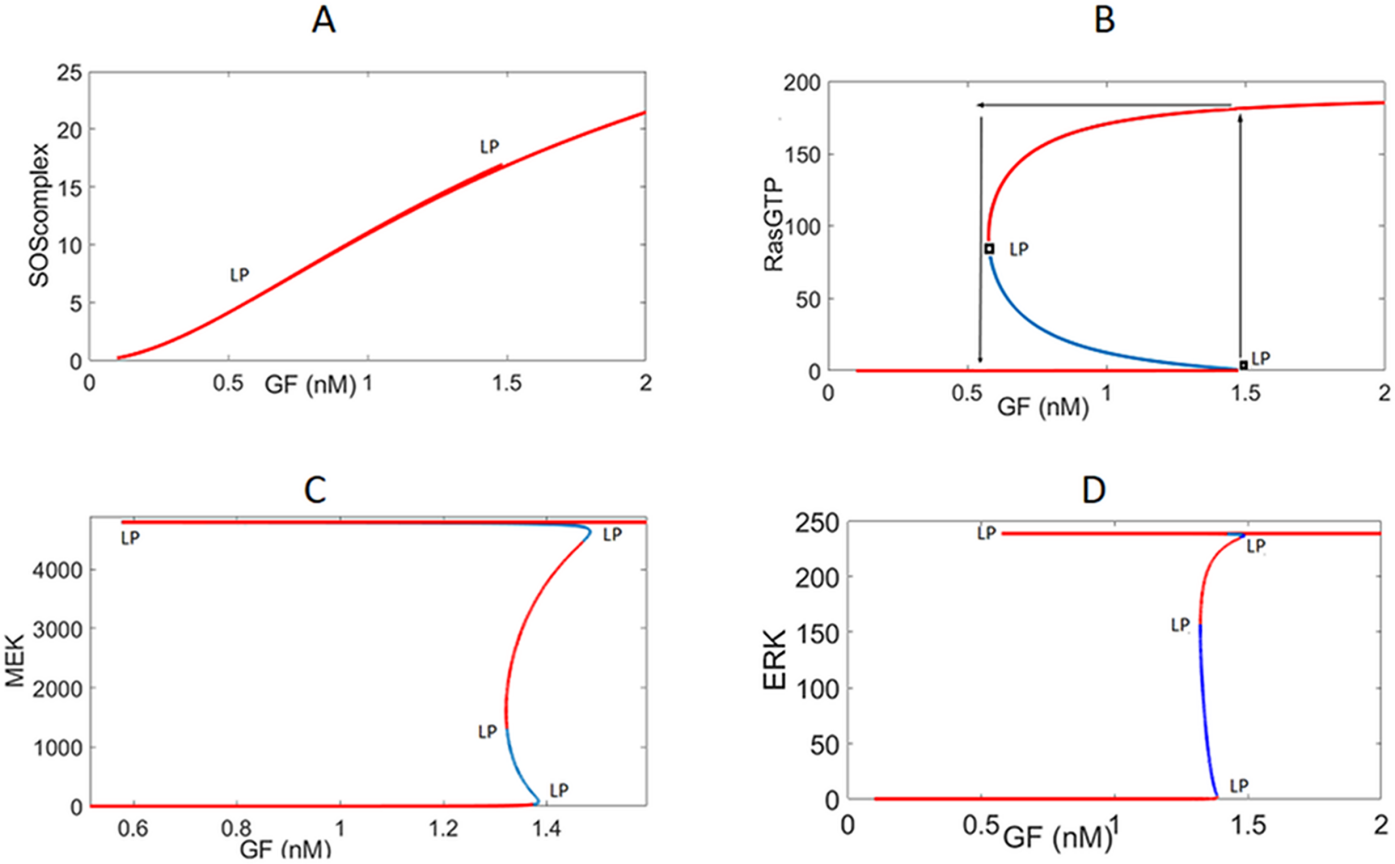
Steady-state response curves without the external feedback loops. GF (growth factor) is the bifurcation parameter that is being changed. Red and blue curves are the stable and unstable branches, respectively. LP: Limit Point bifurcation also called the turning point at which the switch between the low and high stable branches occurs. (A) Since bistability curve is so flat for the ***SOS***_***complex***_, the unstable blue branch is squeezed between the two red stable branches and is not visible. (B) *RasGTP* response. The switch between the low and high stable branches occurs at the turning points, and it is shown by the arrows. (C) Ultrasensitive ***MEK***^***PP***^ response. (D) Bistability is sustained in ***ERK***^***PP***^ response.

Fig 3 shows the steady-state responses of different signaling species as a function of GF. It is seen that the graded dose-response curve of SOS_complex_ is amplified and changed to a *digital* switching response as the signal passes down the pathway. The presence of the internal positive loop IFBL 1 creates an ultrasensitive switching response for RasGTP. This is caused by the bistable response curve of RasGTP which consists of two stable branches separated with an unstable branch. RasGTP can settle on either of these stable branches depending on its initial condition, and it will switch between them as GF is modulated. As Ras switches, it subsequently switches other proteins such as ERK which turns on the transcription factors in the cell cycle to control important cellular functions such as growth and proliferation.

Downstream of RasGTP, MEK^PP^ exhibits another switch-like behavior due to the internal positive feedback loop IFBL2 in the first stage of the MAPK pathway. We also see a drastic increase in the sensitivity (i.e. change in ouput/change in input) of the pathway and amplification of the signal. Interestingly an intermediate stable branch emerges for MEK^PP^. Following

**Fig 4:**
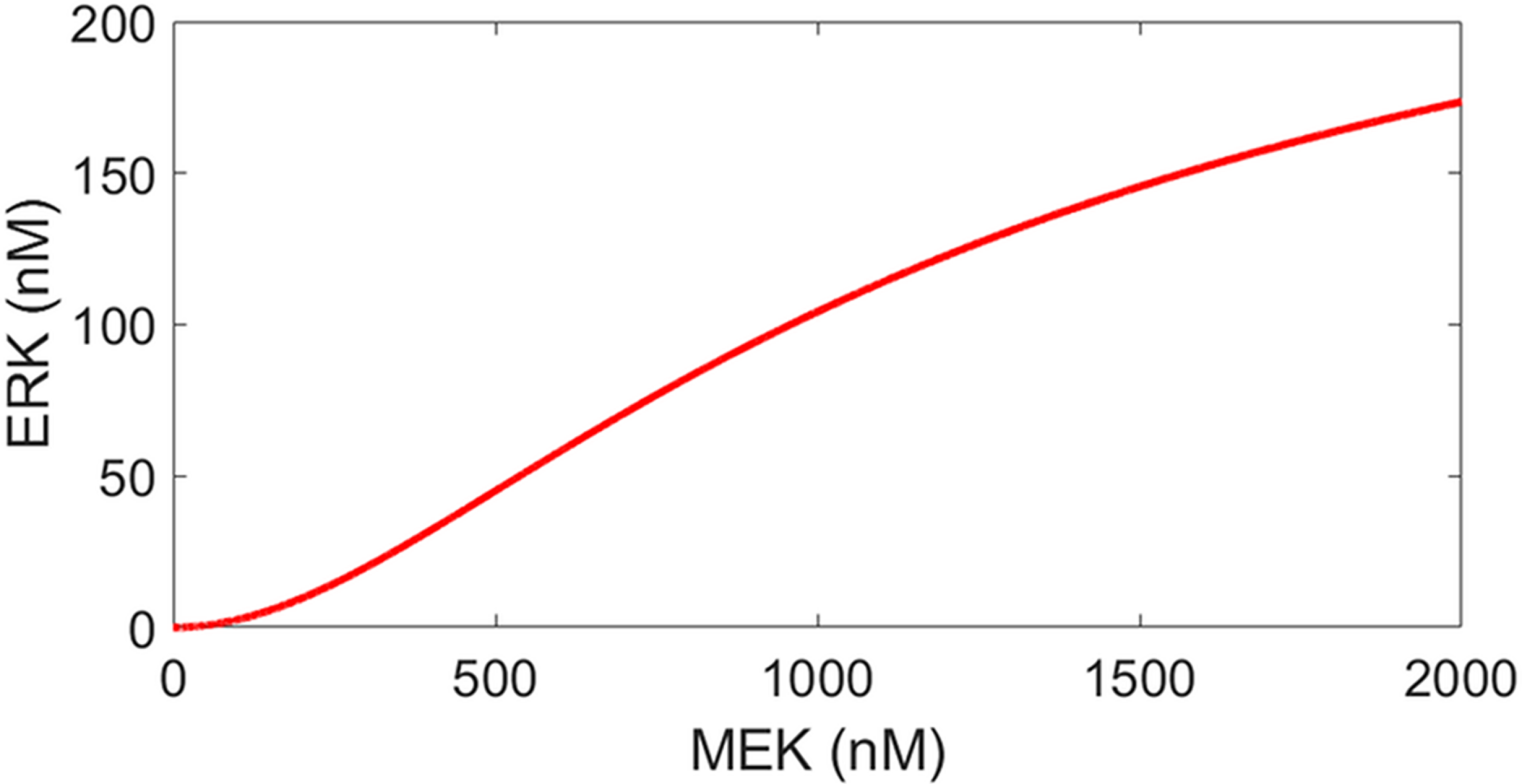
Steady-state response of ERK^PP^ to MEK^PP^.

MEK^PP^, ERK^PP^ shows a similar bistable response as well. However, our calculations have shown that the internal positive feedback loop IFBL3 in the second stage of MAPK pathway is not bistable (see Fig 4). Therefore, the bistable ERK^PP^ response curve is due to the bistable MEK^PP^ response which gets scaled in the second stage of the cascade.

An inherent property of bistable responses is *hysteresis*. In order for ERK to jump from its low state to its high active state, the concentration of GF must exceed its threshold or limit point (LP) of 1.38(nM). However, when ERK becomes activated, it stays active even for GF amounts lower than 1.38 (nM) but greater than 0.58(nM). Further decreasing of GF ligands switches off the system. Thus, for the range 0.58<EGF<1.38(nM), the activity of ERK depends on the previous state of the system because of hysteresis.

**Fig 5:**
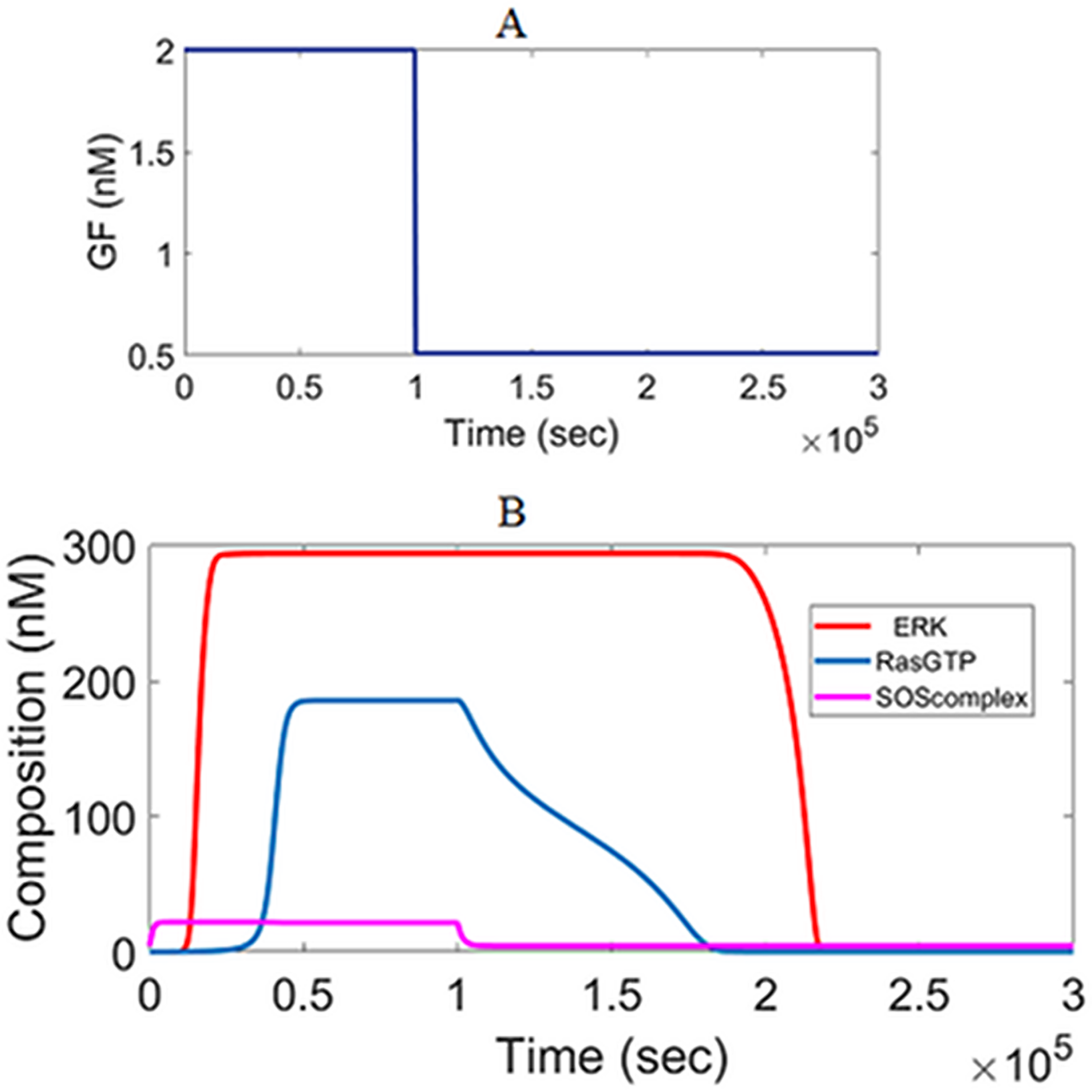
Dynamic simulations showing the transition between the low and high states. (A) Input GF pulse which increases from 0.5 to 2 and returns back to 0.5 nM. (B) Different responses to the input pulse.

Fig 5 shows how the system dynamic responses switch between low and high steady states in response to a GF pulse. Upon stimulus increase, the SOS_complex_ responds first as expected. SOS_complex_ triggers the activation of RasGTP next. This is followed by the dynamic response of ERK^PP^. When the growth factor is withdrawn to its initial value, the system switches back to its lower steady-state.

#### Bistability is robust but can be lost under aberrant conditions

**Fig 6:**
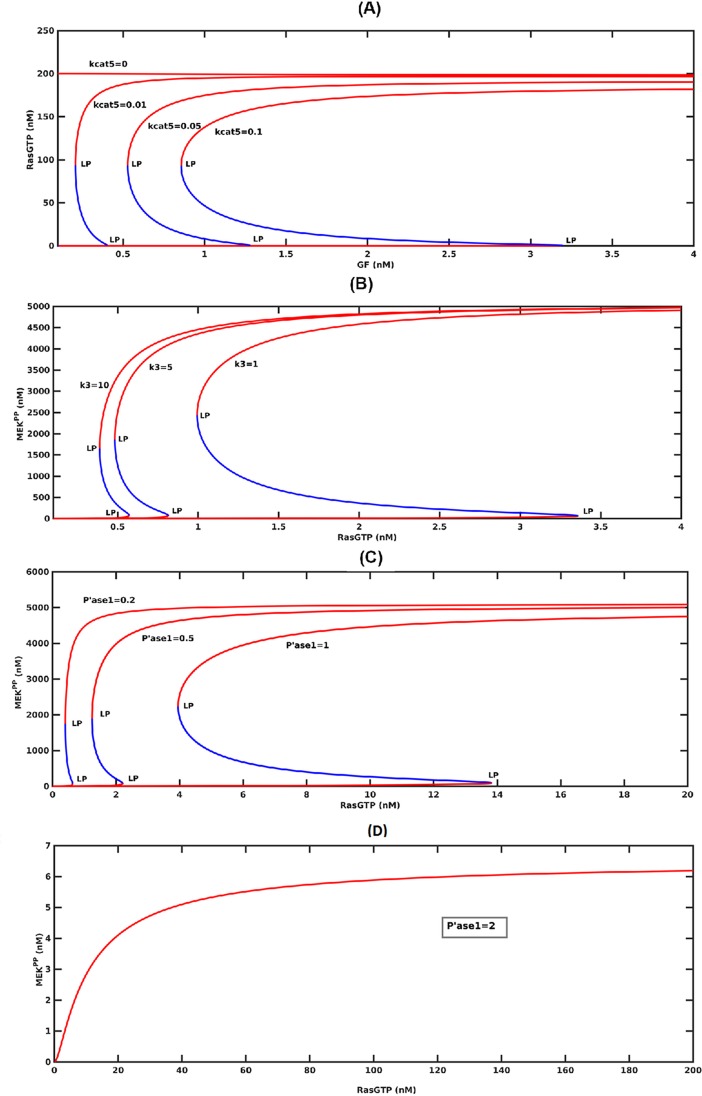
Sensitivity of RasGTP and MEK^PP^ bistability. (A) kcat5 is the rate constant for hydrolysis. (B) Response of the of MAPK pathway when it is disconnected from the rest of the pathway. Sensitivity with respect to k3 which is the rate constant for phoshorylation of MEK. (C) Sensitivity with respect to phosphatase, P’ase 1. (D) Bistability is lost for high levels of phosphatase.

It is known that bistability of biological systems is a robust property i.e. in normal cells it is sustained for significant variations in operating conditions and external perturbations [48] [49]. Otherwise, disease states would emerge. In the context of modeling, this implies that when parameter values and certain inputs are varied within a reasonable range, the model should continue to exhibit bistable behavior. This is illustrated for RasGTP in Fig 6A. Bistability is robust with respect to an order of magnitude change in the rate constant for the hydrolysis of RasGTP to RasGDP, unless hydrolysis is disrupted. It has been reported that mutations decrease the rate of hydrolysis and lock RasGTP in its high state and can cause cancer [50]. The response labeled by kcat5 = 0 in Fig 6A represents this scenario where RasGTP is stuck at its high state and can not switch back to its low state when GF is withdrawn. Similar to the Ras subsystem, bistability of the MAPK subsystem is robust by itself as well. In order to show this, the MAPK subsystem was disconnected from the Ras subsystem and simulated separately by perturbing its parameters. Robustness is shown for one of the important parameters in Fig 6B. **MEK**^**PP**^ response is bistable in the face of variations in k3 (the rate constant for phoshorylation of MEK).

We next consider changes in MEK phosphatase, P’ase 1. Bistability is mantained for a good range of this input as seen in Fig 6C, but further increase beyond a critical threshold (ten-fold the nominal value) results in the loss of bistability as shown by the graded MEK^PP^ response in Fig 6D.

#### Internal negative feedback loop introduces damped oscillations upon GF stimulation

In cells negative feedback is used to combat uncertainty that arises from extracellular fluctuations and internal perturbations [48]. The cooperation between negative and positive feedback loops introduces interesting dynamics and flexibility which is not possible with only one type of feedback loops [51].

**Fig 7:**
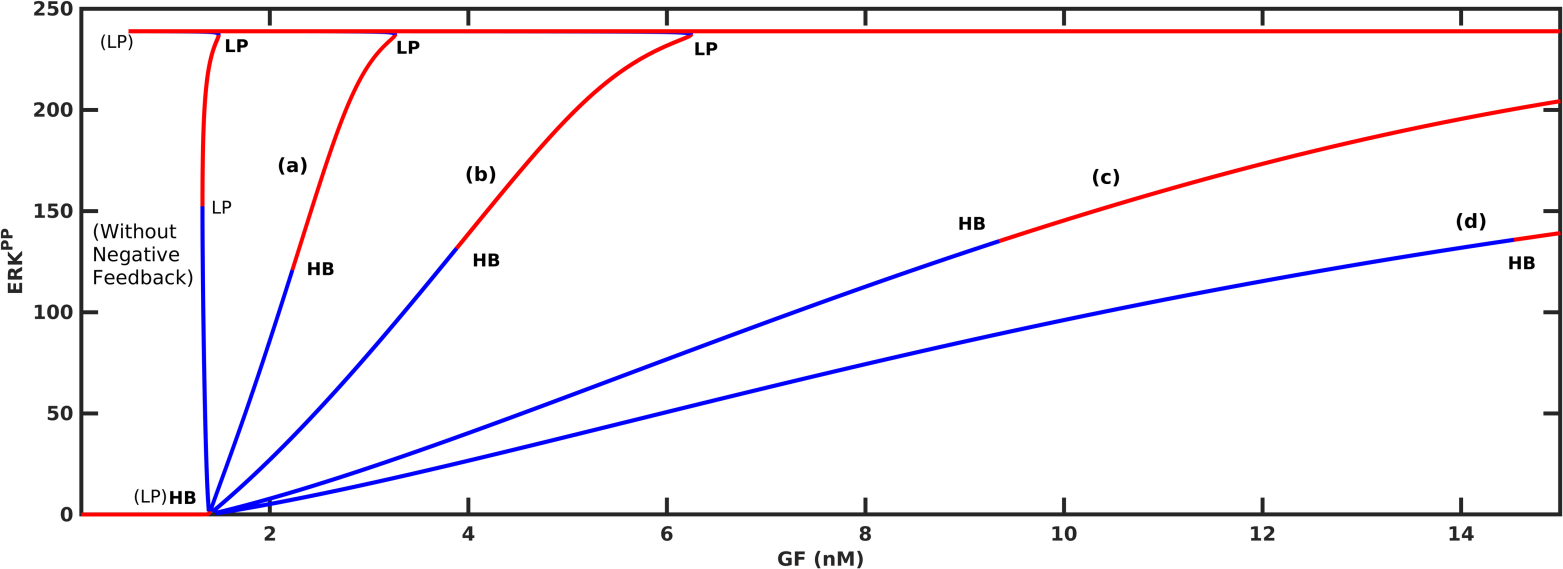
The effect of negative feedback loop FBL4. (a) ***k*_*nfb*_ = 0.03, *Km*_*nfb*_ = 1*e*4.** (b) ***k*_*nfb*_ = 0.1, *Km*_*nfb*_ = 1*e*4.** (c) ***k*_*nfb*_ = 0.03, *Km*_*nfb*_ = 1*e*3.** (d) ***k*_*nfb*_ = 0.1, *Km*_*nfb*_ = 1*e*3.** HB shows the Hopf Bifurcation. In all plots kcat3 = 1.75.

Kholodenko reported that negative feedback can decrease the sensitivity of the MAPK cascade [52]. Our model predictions are in line with these observations. Fig 7 shows that the nonlinear system’s sensitivity (the local slopes on the response curves) decreases when negative feedback is added. Consequently, switching between the low and high stable states requires greater changes in GF concentrations. If the feedback strength is too high, increased disassociation of the SOS_complex_ suppresses the level of ERK activation, and the system is not able to switch within the range of available GF.

**Fig 8:**
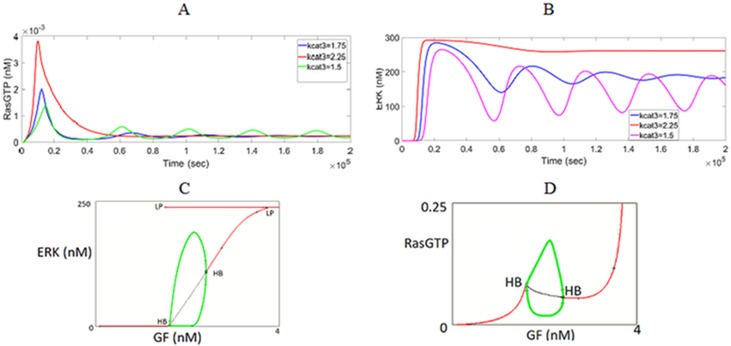
Oscillatory responses. (A) Damped RasGTP oscillations caused by combination of negative and positive feedback loops. GF stimulus is increased from 0.1 to 2.4 (nM). As kcat3 decreases, strength of positive feedback decreases and negative feedback results in more oscillations. (B) Damped ERK oscillations caused by combination of negative and positive feedback loops. (C) Hopf bifurcation and periodic oscillations for ERK^PP^ and (D) for RasGTP. Negative feedback when combined with positive feedback give rise to limit cycles. Green points form the locus of the limit cycles. Four values for GF are considered for dynamic simulations (see Fig **10**). **k**_**nfb**_ **= 0.01 and kcat3 = 1.75**.

It is also theoretically proven that negative feedback can bring out oscillatons in the kinase activities [52]. Shin et al. have treated COS-1 cells with TPA and demonstrated that slow and dampened oscillations in Ras/ ERK activity are caused by negative-feedback inhibition of SOS by ERK [28]. They also showed that this behavior is quite robust to model parameter variations. Nakayama et al. also observed experimentally that ERK activity displayed damped oscillations in FGF-stimulated NIH 3T3 cells [53]. Our model explains these oscillations by the existence of the intermediate stable branch that emerges with negative feedback. Some of the eigenvalues of the steady-states belonging to this branch are complex with negative real parts; therefore, when stimulated by GF, ERK signal settles on this branch after damped oscillations as shown in Fig 8B. In fact it is the relative strenghts of positive and negative feedbacks that determine the existence of oscillations. As kcat3 value decreases, the strength of positive feedback decreases and the negative feedback domination results in more oscillations. When the strength of positive feedback exceeds a threshold and dominates the negative feedback action, ERK response switches to its high stable state without any oscillations.

Nakayama et al. has found out to their surprise that, when NIH 3T3 cells were stimulated with FGF, RasGTP oscillated similar to ERK [53]. They also showed experimentally that ERK-mediated negative feedback phosphorylation of SOS is required for generating Ras oscillations. RasGTP oscillations cannot be predicted by those models in the literature where ERK inhibits the input to the MAPK pathway and not the SOS complex. On the contrary, since ERK inhibits the SOS complex in our model, we can predict the damped oscillations in RasGTP as well (see Fig 8A).

#### Internal positive and negative feedback loops orchestrate coupled relaxation oscillations for RasGTP and ERK

**Fig 9:**
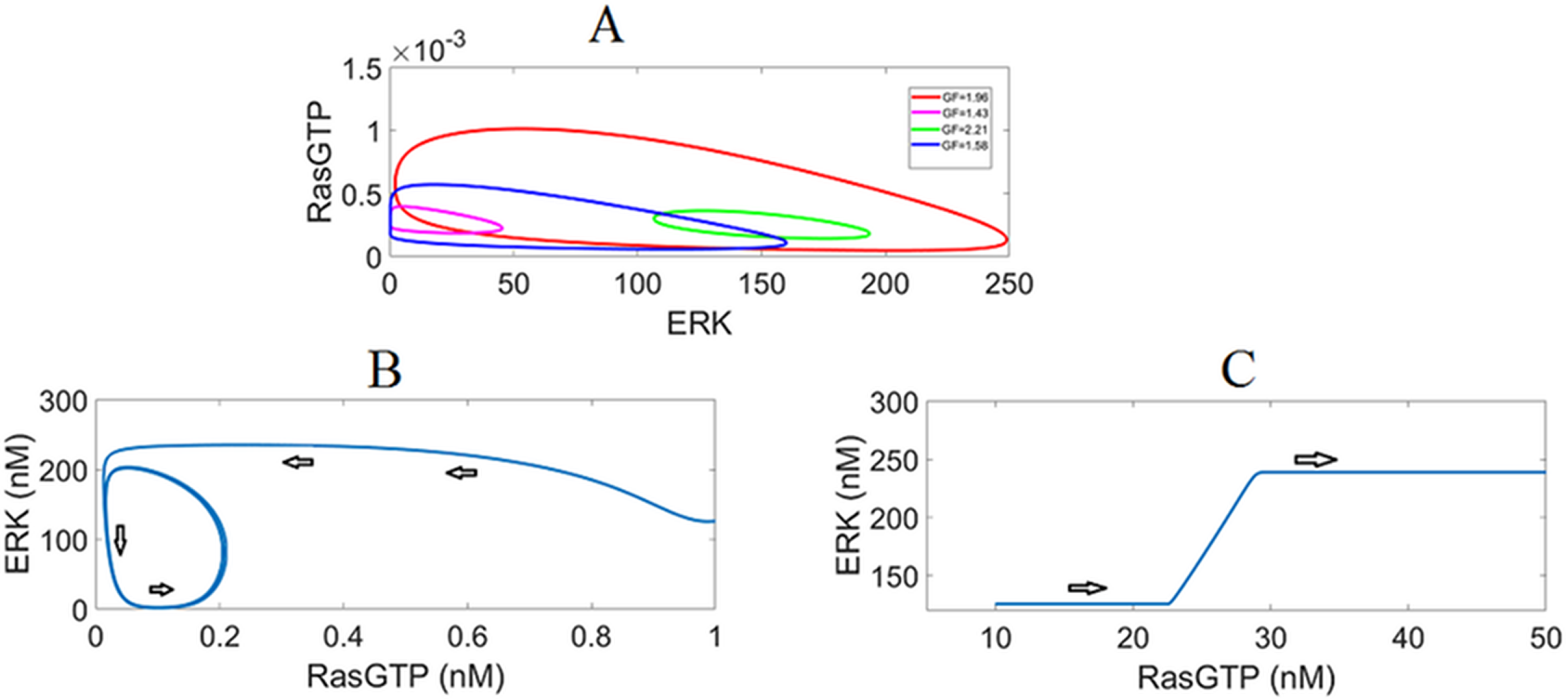
Limit cycles. (A) The limit cycles in phase-plane. (B) The response starting from a lower value of RasGTP converges to the limit cycle indicated by the spiral trajectory. (C) The response starting from a high RasGTP value converges to the stable non-oscillatory high state.

**Fig 10:**
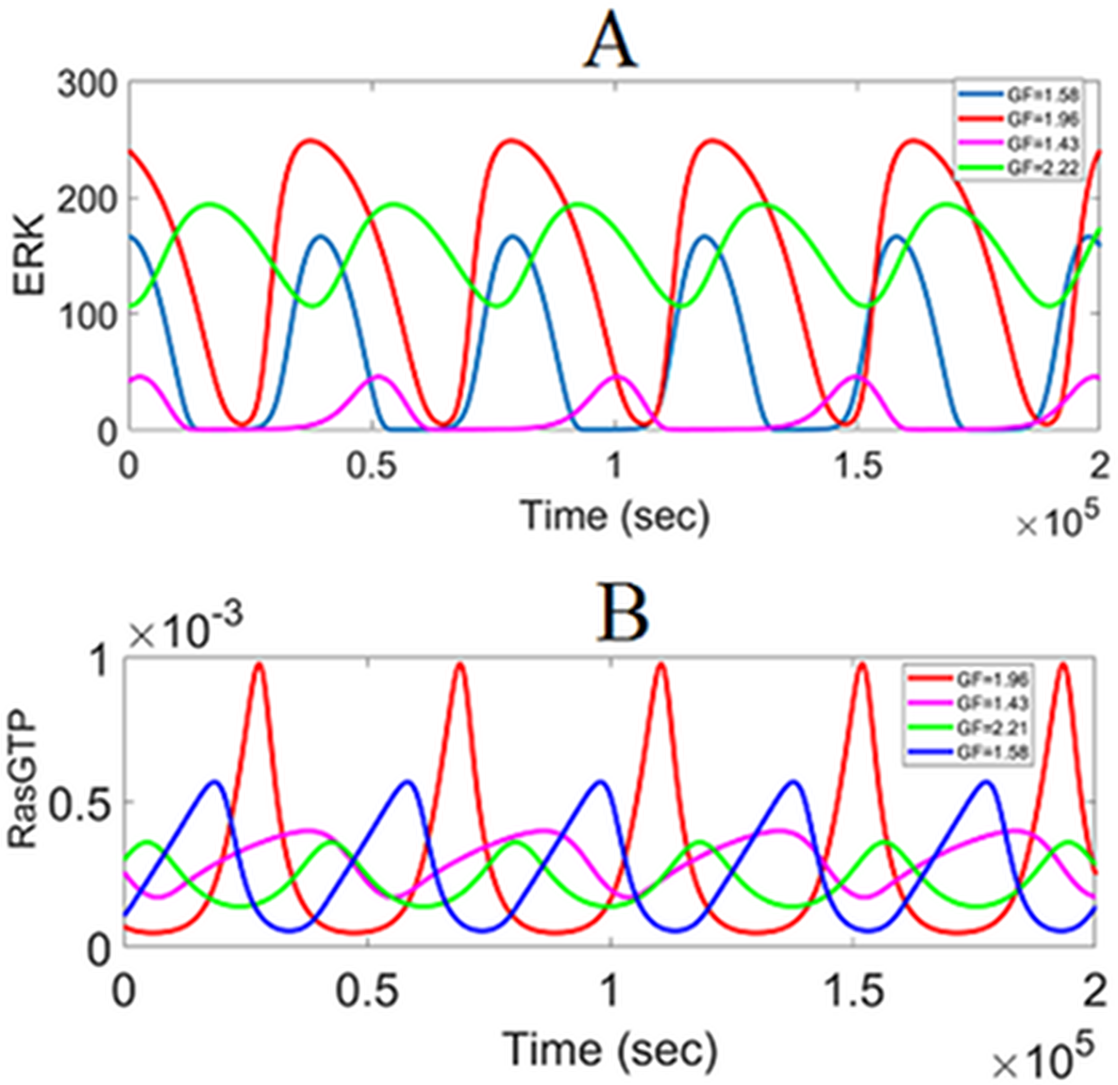
Dynamics of periodic oscillations. (A) ERK^PP^ oscillations. (B) RasGTP oscillations.

The other important change introduced by the negative feedback loop IFBL4 is the appearance of *HB (Hopf bifurcation)* points as illustrated in Fig 8C and 8D. Sustained periodic solutions emerge from these points resulting in limit cycles (Fig 9A). It is worth mentioning that these oscillations occur in the absence of a time delay. In this regime, the negative feedback is strong enough to make the system switch back and forth between the discrete stable states generated by the positive feedback loop, leading to sharp “pulse-like” oscillations (Fig 10A and 10B). This kind of oscillatory behavior is also known as *relaxation oscillations* that exhibit different timescales, with slow negative feedback operating over fast positive feedback [54][55][56][57]. In our case RasGTP and ERK oscillations are coupled. Mechanistically speaking, when ERK switches sharply to its high state upon GF stimulation, it inhibits the SOS complex and RasGTP switches to its low state. This in turn brings ERK to its low state; the SOS complex is less inhibited; RasGTP increases and oscillations complete the cycle. When ERK oscillations are near their max(s), RasGTP oscillations are near their min(s) as implied by comparing Fig 10A and 10B.

Historically, persistent oscillations have been reported for the MAPK pathways [57][58][59]. Waters et al. have shown that transforming growth factor, TGFα, induces persistent ERK oscillations in human keratinocytes [58]. They showed that MED1 gene was activated by ERK oscillations which could impact a broad network of transcription factors. It was also reported that the ERK cascade shows very robust oscillatory behavior in human mammary epithelial cells (HMECs) in the presence of EGF [59]. Relaxation oscillations predicted by our model and the underlying mechanism are similar to the work of Kochańczyk et al. [57]. In [57] the positive feedback from Ras to SOS is coupled with the negative feedback loop from ERK to SOS, and the system produces relaxation oscillations which match experimental time courses.

Fig 10 shows the dose (GF) dependency of oscillations. Waveforms with different amplitudes and periods can be realized depending on the level of growth factor. Also, the responses are sensitive to the initial conditions due to the nonlinear dynamics as seen in Fig 9B and 9C. Depending on the initial RasGTP and ERK concentrations, the responses can either converge to the nearest limit cycle or either of the two stable steady-states (low or high).

After we establish the underlying dynamics of the signaling pathway with its built-in internal feedback loops, we next study how each external feedback loop modulates the ERK signal.

#### External autocrine positive feedback loop sustains ERK activity for lower GF levels, but excessive feedback action results in one-way toggle switch

**Fig 11:**
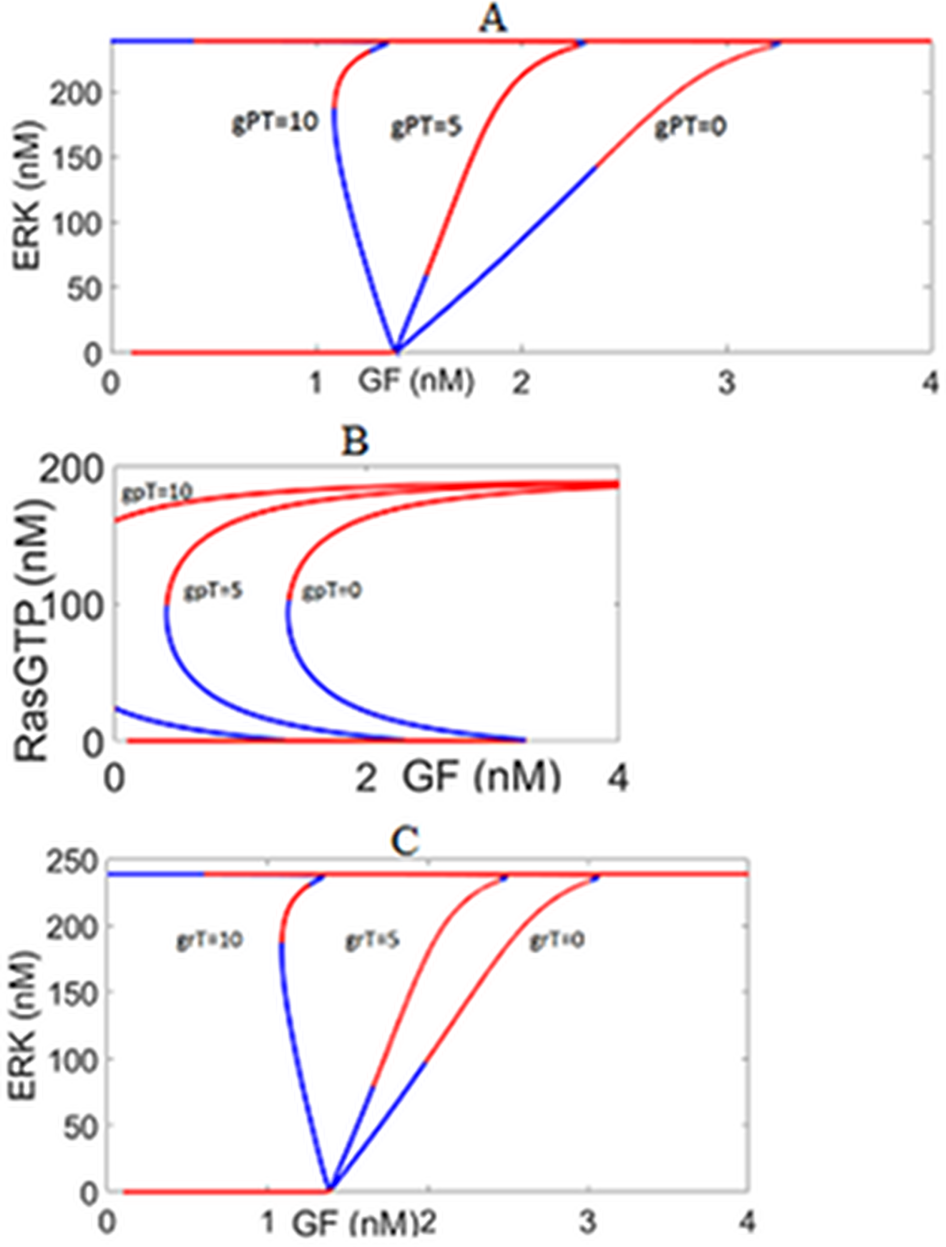
The impact of the autocrine positive feedback loop EFBL1. (A) ERK response to GF for different gpT values. (B) RasGTP response to GF for different gpT values. (C) ERK response to GF for different grT values. The feedback strength is represented by gpT. The linear gain in ligand production is denoted by grT. Less GF ligand is required to switch on ERK and Ras, and more ligands need to be removed to switch off the active Ras and ERK. For gpT = 10 or grT = 10, LP appears at negative GF, suggesting that once ERK and Ras are active, they cannot be switched off. knfb = 0.01, kcat3 = 1.75.

Bistable switching responses generated by the internal positive feedback loops are maintained when the autocrine loop is added as shown in Fig 11. At the same time, less amount of fresh extracellular GF is required to switch on ERK^PP^ since TACE releases some of the membrane bound ligands and makes them available for signaling. This is illustrated by the response curves shifting to lower GF values as the strength of positive feedback and the ligand production increases. When the autocrine action exceeds a certain threshold, Ras and ERK activities can switch only in one direction i.e. from low to high and they persistently remain active, even after removing all of the GF ligands. This potentially leads to the cancerous signaling molecules [5].

#### External negative feedback loop due to Argos inhibits the activation of ERK and introduces additional HB points and oscillations

**Fig 12:**
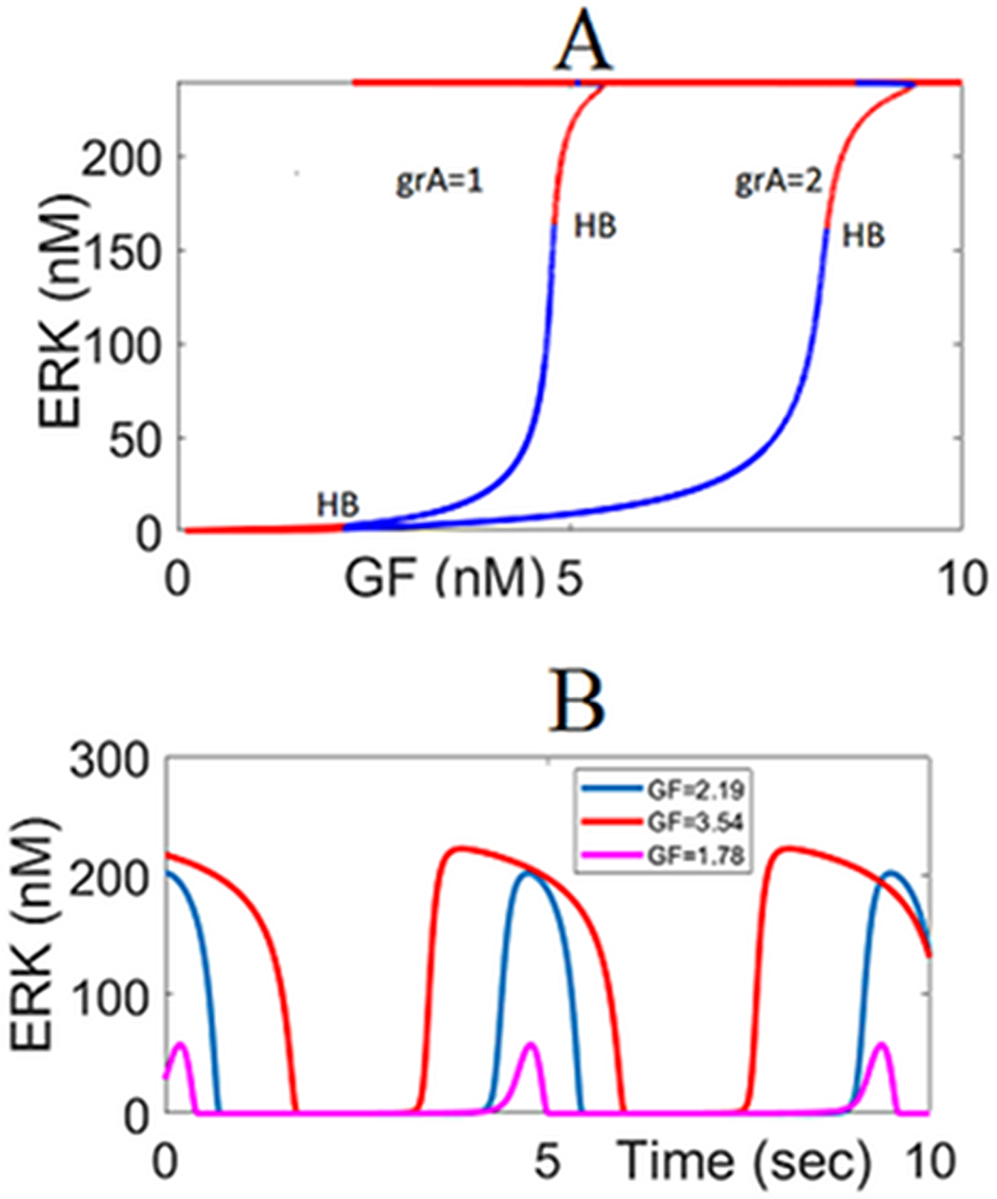
The impact of the negative feedback loop EFBL2. (A) *ERK*^*PP*^ response to GF. Argos introduces two new HB points. knfb = 0.0, kcat3 = 1.75 (B) Oscillations emanate from the HB points and their amplitudes depend on the GF. grA = 1.

In Fig 12 it is shown that more GF is required to maintain the same level of ERK activation when the negative feedback loop EFBL2 is active. EFBL2 manipulates ARGOS which binds to EGFR and inhibits its binding with GF; thus, suppresses the ERK signal. Two HB points emerge due to the negative feedback loop EFBL2. Depending on the dose of the growth factor, these new HB points create different amplitude oscillations for RasGTP and ERK^PP^.

#### The external feedback loops can generate the necessary ERK dynamics to control the cell cycle

**Fig 13:**
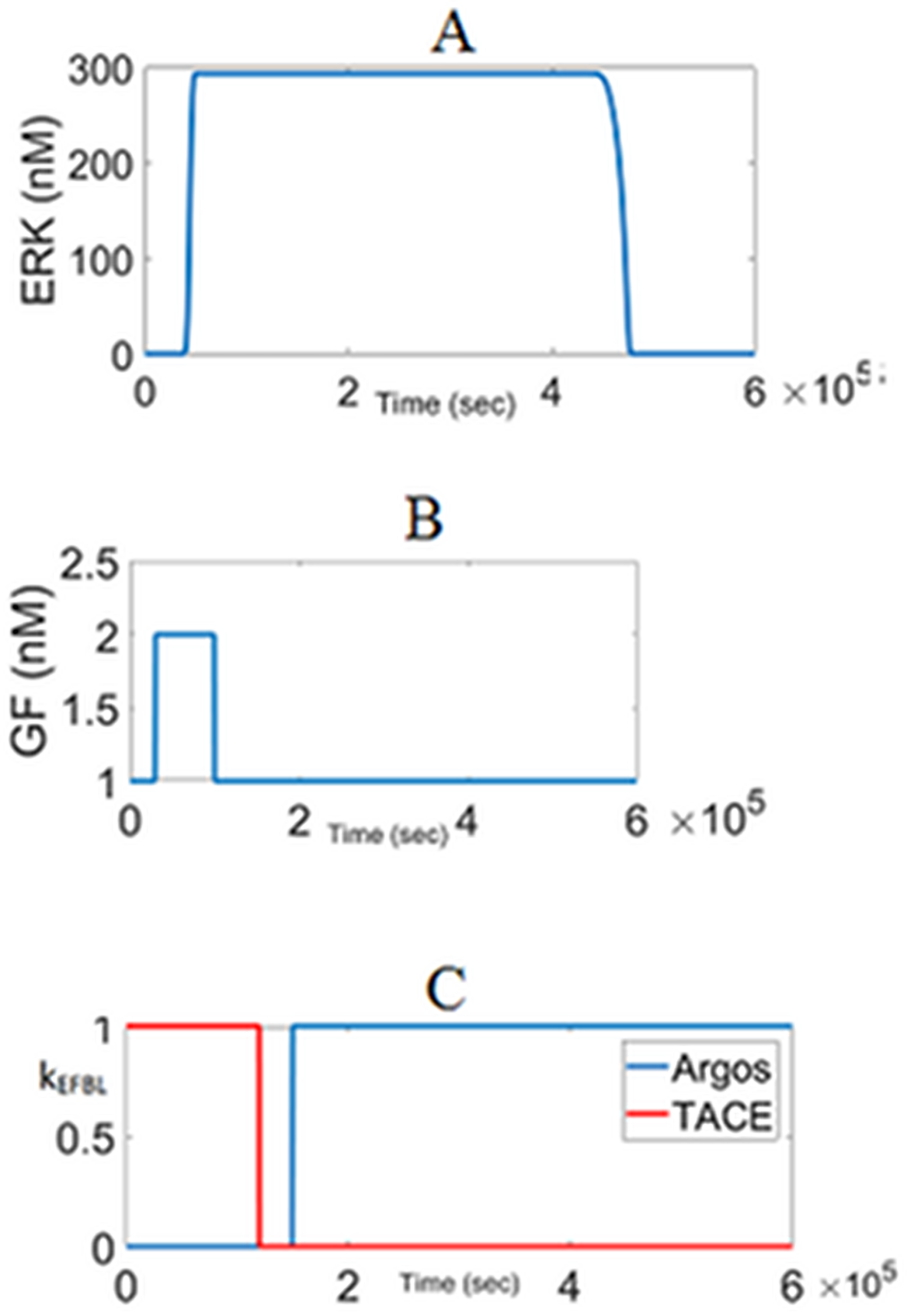
Generation of a desired ERK response profile by the external feedback loops. (A) ERK^PP^ response. (B) GF pulse. (C) External feedback loops EFBL1 and EBFL2. k_EFBL_ = 1 indicates that the loop is on and k_EFBL_ = 0 indicates it is off. kcat3 = 1.75, gpT = 10, knfb = 0.0. grA = 1.

The ERK response profile shown in Fig 13A is typical of those seen during cell cycle control [60]. Upon GF stimulation, ERK switches to ON state and ERK^PP^ translocates to the nucleus, where it phosphorylates several nuclear transcription factors, that govern cellular responses [16][17]. Phosphorylated transcription factors stimulate transcription of genes which are responsible for cell cycle progression (e.g. cyclins) [17]. Thus, when ERK is switched on, cyclin D is synthesized, and the cell cycle enters the growth phase G_1_. This is followed by the transcription of cyclin E and entry into the synthesis phase, S, of the cell cycle. Once the cell commits to synthesis, growth factor is no longer needed. Decrease in GF results in the inactivation of ERK (i.e. switch to Off state) and a drop in cyclin D.

The switching times of ERK (i.e. the times at which ERK switches ON and OFF) and the signal duration (the length of time ERK stays active) can be modulated by GF and the external feedback loops due to TACE and Argos. In fact, the external feedback loops EFBL1 and EFBL2 operate together as a split-range controller [61]. In a split-range controller, a single output (ERK) is controlled by manipulating two inputs (TACE and Argos). While TACE provides persistent activation, Argos tunes down the ERK signaling when needed. By utilizing this two-degree of freedom, external feedback loops EFBL1 and EFBL2 can produce a desired ERK^PP^ dynamics under a wide range of conditions. This is illustrated in Fig 13. At the beginning of simulation EFBL1 is on, EFBL2 is off. GF changes as a pulse to initiate the signaling. In response ERK^PP^ switches to its active state. Activity of ERK^PP^ is sustained by EFBL even when GF returns back to its initial value. When the desired duration of ERK^PP^ is over, positive EFBL1 is turned off and negative EFBL2 is turned on. As a result, ERK^PP^ switches back to its original inactive state.

## Conclusions

In this work we have modeled and analyzed the ERK signaling by decomposing its pathway into three distinct subsystems that were named after the SOS complex, RasGTP and MAPK. These subsystems were shown to interact through several feedback loops which resulted in a diverse array of dynamics for the signaling proteins in the network in general, and RasGTP and ERK in particular. The dynamic model was constructed by integrating the mechanistic models of the individual subsystems of the signaling pathway and of the feedback controllers. The model consists 46 ordinary differential equations and 17 algebraic equations. Parameter values were taken from the literature and very few of them had to be retuned to match the well-established qualitative behaviors reported in the literature.

In our model and in its subsequent analysis, we have categorized the feedback loops into internal (those embedded into the kinetics of the pathway) and external (those that use actuating species like TACE and ARGOS that act from outside the main pathway) loops. It was shown that combination of positive and negative feedback loops modulates the ERK dynamics (duration, amplitude, frequency, oscillations, stability) selectively to obtain the specific biological outcome.

Through bifurcation analysis of the developed model, we have shown that internal positive feedback loops within the Ras and MAPK subsystems are responsible for the existence of bistability and switching dynamics. Addition of internal negative feedback from ERK to the SOS complex, ShC-Grb2-SOS, introduces either damped or sustained oscillatory responses depending on the amount of growth factor. External inhibitory feedback due to ARGOS creates additional periodic orbits that are dependent on the GF levels. The other external autocrine positive feedback loop due to TACE sustains ERK activity for lower GF levels, but excessive feedback action results in one-way toggle switch.

The proposed autocrine feedback model does not consider the diffusion effects since our major objective was to assess the overall effect of the autocrine feedback loop on ERK dynamics which we did not want to complicate with the potential difficulties that would be introduced by the reaction-diffusion models. In future studies, the existing reaction-diffusion models for autocrine systems [44, 62] can be combined with our proposed signaling model.

Finally, the model is able to explain the experimental data and observations reported on the literature. The modular structure of the model makes it possible to include new interactions and feedback loops, if needed. It can be used to test and design different control mechanisms and to generate new hypothesis for further validation.

## Supporting information

S1 Fig Effects of variations in the value of K5m. (TIF)

S2 Fig Effects of variations in the value of kcat3. (TIF)

S3 Fig The bistable regime for RasGTP after parameter tuning. kcat3 = 1.75s-1 and K5m = 18 nM.
(TIF)

S4 Fig Effects of variations in the value of RGAP. (TIF)

S5 Fig Effects of *β* on the bistable regime. (TIF)

S1 Text The GF-SOS subsystem model. (DOCX)

S2 Text The Ras subsystem model. (DOCX)

S3 Text The MAPK subsystem model. (DOCX)

S4 Text Parameters and reactions of the external feedback loops. (DOCX)

S5 Text Model equations. (DOCX)

S6 Text XPPAUT file of the model. (TXT)
