# Supplementary figures and images for: Dynamics and control of the ERK signaling pathway: Sensitivity, bistability, and oscillations
Source: PLoS One. 2018 Apr 9;13(4):e0195513. doi: 10.1371/journal.pone.0195513 (PMC5891012; doi:10.1371/journal.pone.0195513)

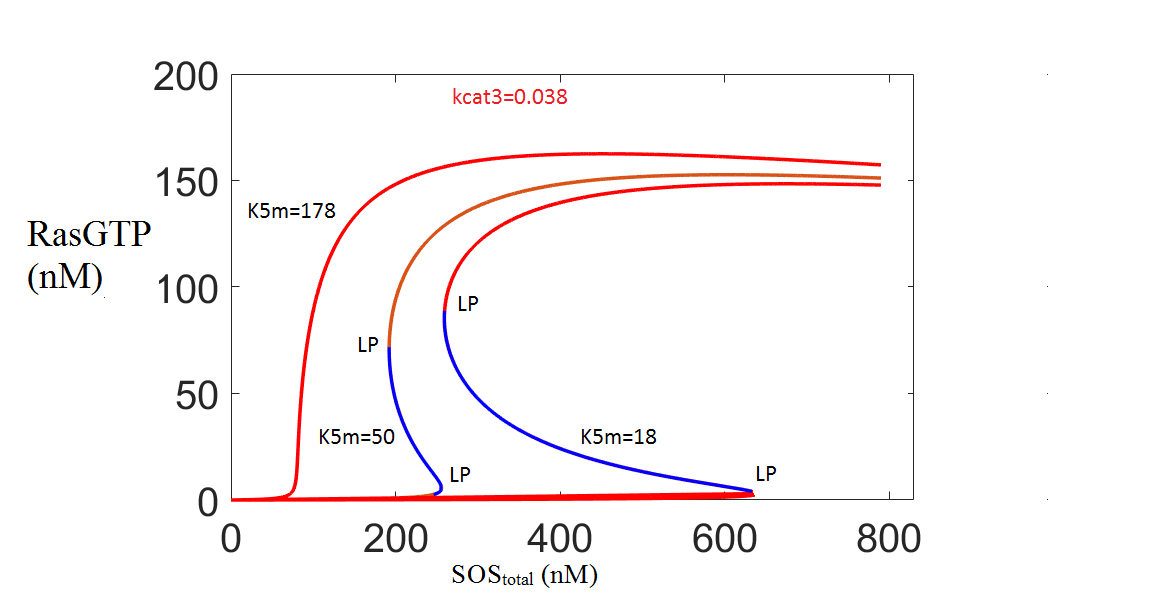

Supplement: S1 Fig — (TIF) [file pone.0195513.s001.tif]

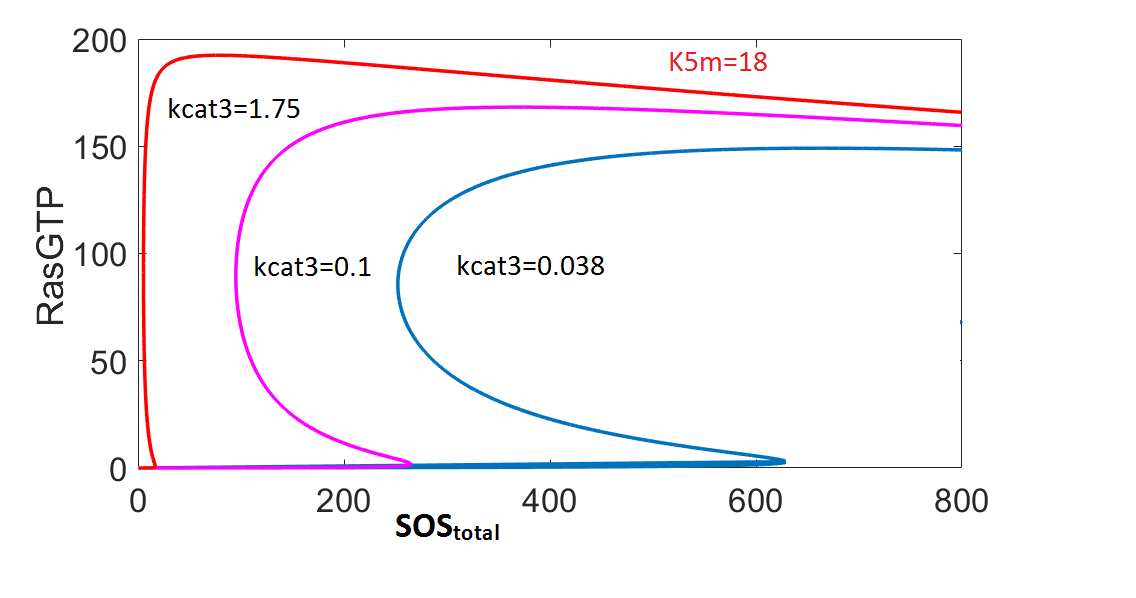

Supplement: S2 Fig — (TIF) [file pone.0195513.s002.tif]

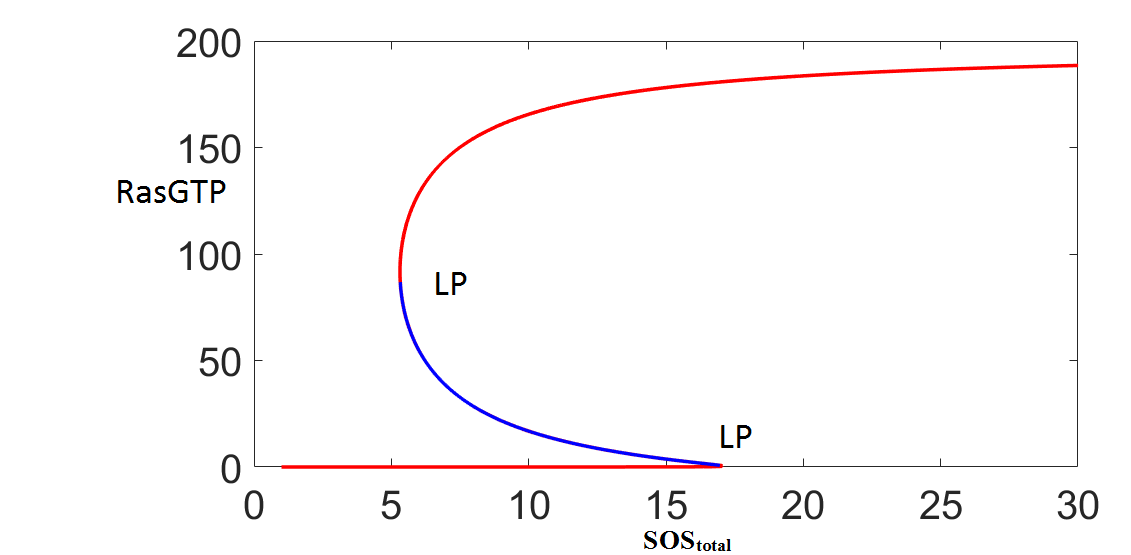

Supplement: S3 Fig — kcat3 = 1.75s-1 and K5m = 18 nM. (TIF) [file pone.0195513.s003.tif]

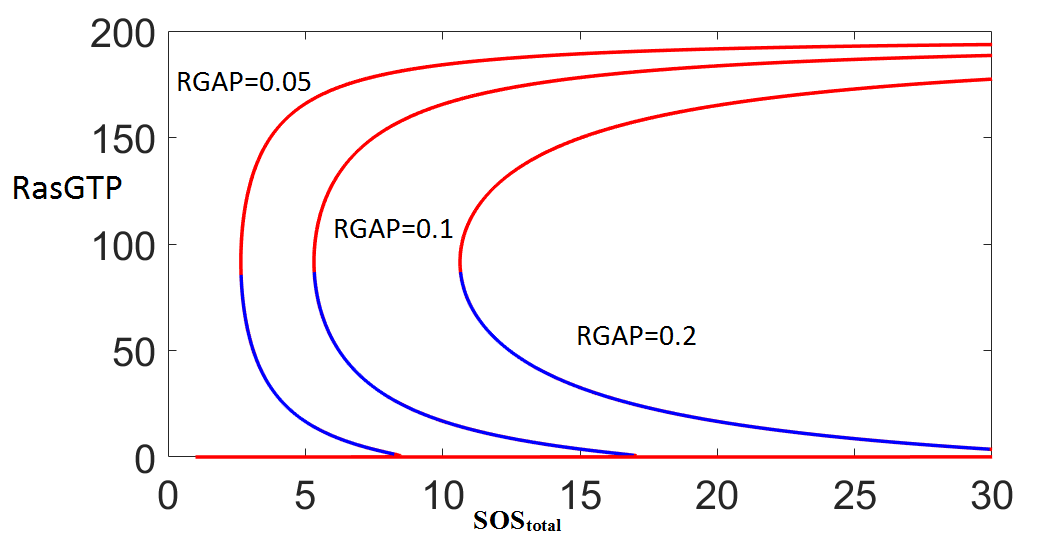

Supplement: S4 Fig — (TIF) [file pone.0195513.s004.tif]

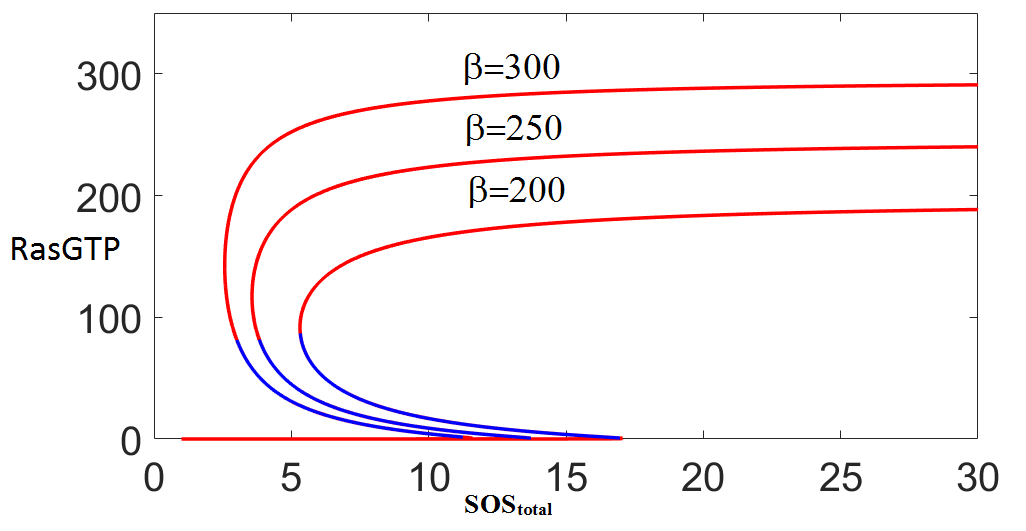

Supplement: S5 Fig — (TIF) [file pone.0195513.s005.tif]
