# Supplementary material for: Dynamics and control of the ERK signaling pathway: Sensitivity, bistability, and oscillations
Source: PLoS One. 2018 Apr 9;13(4):e0195513. doi: 10.1371/journal.pone.0195513 (PMC5891012; doi:10.1371/journal.pone.0195513)
Supplement: S1 Text — (DOCX) [file pone.0195513.s006.docx]

**S1 Text. The GF-SOS subsystem model.**

Kholodenko et al. [1] derived a kinetic model of EGFR signaling pathway to describe the complex cellular responses to EGF. We have adopted this model for our GF-SOS subsystem. The rate equations are the parameter values are listed in Table S1.

Table S1- Parameters and rate equations for the SOS complex model.

| **Reaction**  **No.** | **Parameter value** | **Rate equations** |
| --- | --- | --- |
| **1** | kf1=0.003, kb1=0.06 | kf1*[EGFR]*[EGF]-kb1*[EGF_EGFR] |
| **2** | kf2=0.01, kb2=0.1 | kf2*[EGF_EGFR]*[EGF_EGFR]-kb2*[(EGF_EGFR)2] |
| **3** | kf3=1, kb3=0.01 | kf3*[(EGF_EGFR)2]-kb3*[(EGF_EGFR)2-P] |
| **4** | Vu4=450, Ku4=50 | Vu4*[(EGF_EGFR)2-P]/(Ku4+[(EGF_EGFR)2-P]) |
| **5** | kf5=0.06, kb5=0.2 | kf5*[(EGF_EGFR)2-P]*[PLCg]-kb5*[(EGF_EGFR)2_PLCg] |
| **6** | kf6=1, kb6=0.05 | kf6*[(EGF_EGFR)2_PLCg]-kb6*[(EGF_EGFR)2_PLCg-P] |
| **7** | kf7=0.3, kb7=0.006 | kf7*[(EGF_EGFR)2_PLCg-P]  -kb7*[(EGF_EGFR)2-P]*[PLCg-P] |
| **8** | Vu8=1, Ku8=100 | Vu8*[PLCg-P]/(Ku8+[PLCg-P]) |
| **9** | kf9=0.003, kb9=0.05 | kf9*[(EGF_EGFR)2-P]*[Grb2]-kb9*[(EGF_EGFR)2_Grb2] |
| **10** | kf10=0.01, kb10=0.06 | kf10*[(EGF_EGFR)2_Grb2]*[SOS]  -kb10*[(EGF_EGFR)2_Grb2_SOS] |
| **11** | kf11=0.03, kb11=4.5e-3 | kf11*[(EGF_EGFR)2_Grb2_SOS]  -kb11*[(EGF_EGFR)2-P]*[Grb2_SOS] |
| **12** | kf12=1.5e-3, kb12=1e-4 | kf12*[Grb2_SOS]-kb12*[Grb2]*[SOS] |
| **13** | kf13=0.09, kb13=0.6 | kf13*[(EGF_EGFR)2-P]*[Shc]-kb13*[(EGF_EGFR)2_Shc] |
| **14** | kf14=6, kb14=0.06 | kf14*[(EGF_EGFR)2_Shc]-kb14*[(EGF_EGFR)2_Shc-P] |
| **15** | kf15=0.3, kb15=9e-4 | kf15*[(EGF_EGFR)2_Shc-P]  -kb15*[Shc-P]*[(EGF_EGFR)2-P] |
| **16** | Vu16=1.7, Ku16=340 | Vu16*[Shc-P]/(Ku16+[Shc-P]) |
| **17** | kf17=0.003, kb17=0.1 | kf17*[(EGF_EGFR)2_Shc-P]*[Grb2]  -kb17*[(EGF_EGFR)2_Shc_Grb2] |
| **18** | kf18=0.3, kb18=9e-4 | kf18*[(EGF_EGFR)2_Shc_Grb2]  -kb18*[(EGF_EGFR)2-P]*[Shc_Grb2] |
| **19** | kf19=0.01, kb19=2.14e-2 | kf19*[(EGF_EGFR)2_Shc_Grb2]*[SOS]  -kb19*[(EGF_EGFR)2_Shc_Grb2_Sos] |
| **20** | kf20=0.12, kb20=2.4e-4 | kf20*[(EGF_EGFR)2_Shc_Grb2_Sos]  -kb20*[Shc_Grb2_SOS]*[(EGF_EGFR)2-P] |
| **21** | kf21=0.003, kb21=0.1 | kf21*[Shc-P*Grb2]-kb21*[Shc_Grb2] |
| **22** | kf22=0.03, kb22=0.064 | kf22*[Shc_Grb2]*[SOS]-kb22*[Shc_Grb2_SOS] |
| **23** | kf23=0.1, kb23=0.021 | kf23*[Shc_Grb2_SOS]-kb23*[Shc-P*Grb2_SOS] |
| **24** | kf24=0.009, kb24=4.29e-2 | kf24*[(EGF_EGFR)2_Shc-P]*[Grb2_SOS]  -kb24*[(EGF_EGFR)2_Shc_Grb2_Sos] |
| **25** | kf25=1, kb25=0.03 | kf25*[PLCg-P]-kb25*[PLCg-P-I] |
| **Conserved moieties (nM)** | | |
| [EGFR _tot_] = 10, [EGF _tot_]= 5, [PLCγ _tot_]= 105, [Grb2 _tot_]= 85, [Shc _tot_]= 150, [SOS _tot_]= 50. | | |

**Reference:**

[1] B. N. B. N. Kholodenko, O. O. V Demin, G. Moehren, and J. B. Hoek, “Quantification of Short Term Signaling by the Epidermal Growth Factor Receptor *,” *J. Biol. Chem.*, vol. 274, no. 42, pp. 30169–30181, 1999.
