# Supplementary material for: Dynamics and control of the ERK signaling pathway: Sensitivity, bistability, and oscillations
Source: PLoS One. 2018 Apr 9;13(4):e0195513. doi: 10.1371/journal.pone.0195513 (PMC5891012; doi:10.1371/journal.pone.0195513)
Supplement: S2 Text — (DOCX) [file pone.0195513.s007.docx]

**S2 Text. The RAS subsystem model.**

For the RAS subsytem we adopted the model developed by Das et al. [1]. This model includes the SOS-mediated allosteric activation of RAS and its deactivation by RasGAP. In order to be consistent with the concentrations in the GF-SOS and MAPK subsystem models, we changed the concentration unit given as $molecules/({\mu m)}^{3}$in [1] to *nano-molar* (nM). The nominal kinetic parameters given in Table 1 in the original article [1] were converted to the new values shown in Table S2 below:

Table S2- Parameters and reactions for the Ras subsystem [1].

| **Index** | | **Parameter values** |  |
| --- | --- | --- | --- |
| **1** | kfi1=1.1e-4(nM)^-1^s^-1^ kbi1=3s^-1^ | 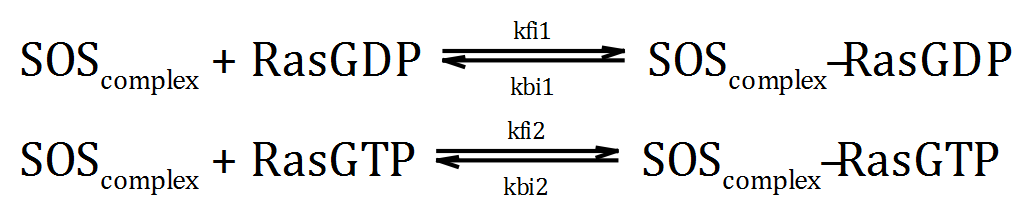 | |
| **2** | kfi2=1.0e-4(nM)^-1^s^-1^ kbi2=0.4s^-1^ | 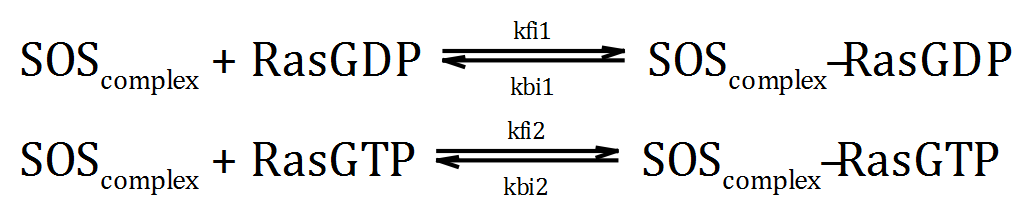 | |
| **3** | kcati3=0.38s^-1^  Ki3m=2738(nM) | 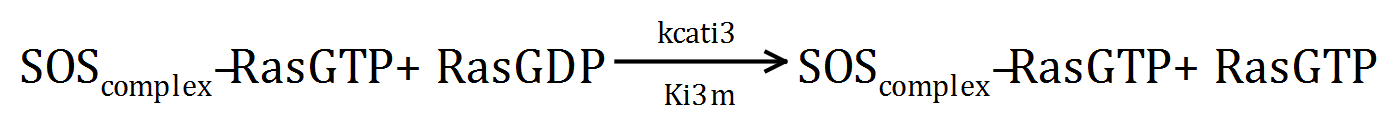 | |
| **4** | kcati4=0.003s^-1^  Ki4m=15230(nM) | 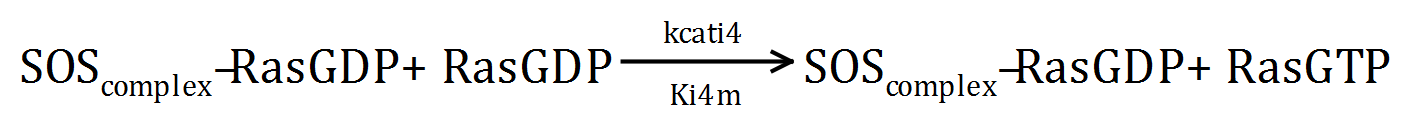 | |
| **5** | kcati5=0.1s^-1^  Ki5m=178(nM) | 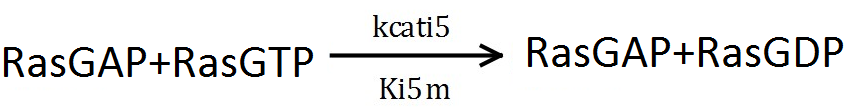 | |
| **Conserved moieties** (nM) | | | |
| $\alpha=\left[ SOS_{complex} \right]+\left[ SOS_{complex}-RasGDP \right]+\left[ SOS_{complex}-RasGTP \right]$  $\beta=\left[ RasGDP \right]+\left[ RasGTP \right]+\left[ SOS_{complex}-RasGDP \right]+[SOS_{complex}-RasGTP]$ | | | |

Since the exact values are not known for most of the parameters, the authors in [1] have done a sensitivity analysis and shown that bistability and hysteresis hold for a wide range of parameter values.

In our model the input to the RAS subsystem is the $SOS_{complex}$ from the upstream GF-SOS subsystem. The output of the RAS subsystem model is RasGTP which triggers the ERK signaling by activating the downstream MAPK subsystem. It is well-known that RasGTP exhibits a bistable response which provides the gate switch for the propagation of the signal from the growth factor to ERK. Therefore, when connecting the RAS model to the GF-SOS subsystem, some of its parameters had to be adjusted to assure that RasGTP maintains its bistable response with hysteresis for the range of the $SOS_{complex}$ concentrations provided by the upstream GF-SOS subsystem. We chose to tune the rate constant kcat3 and the Michealis constant K5m which affect the allosteric activation and deactivation of RasGTP, the two most important reactions that control the strength of positive feedback and bistability. Since there is uncertainty about the values of these parameters, they were also adjusted in [1] around their nominal values.

1. **Effect of K5m**

Here we fix all the kinetic parameters except K5m to their nominal values given in Table S2. The total amount of SOS, $\alpha,$ is treated as the bifurcation parameter.

For the base case the following values were assigned to the concentrations: $\beta=200 nM;RGAP=0.1 nM.$ S1 Fig. shows how kcat3 affects RasGTP response under these conditions.

**S1 Fig.** Effects of variations in the value of K5m.

As K5m decreases, the response curves shift to the right since deactivation increases and more SOS is required for the activation of Ras. The result is similar to the one observed in [1] (see Fig.S3 in [1]). The response curve for K5m=18 provides a large jump in Ras activity above a threshold value of SOS_total_=600 nM which is indicative of the allosteric activation. Therefore we choose K5m=18 for the subsequent analysis.

1. **Effect of k3cat**

S2 Fig. shows that as the value of k3cat increases, the bistable regime shifts to the lower SOS values since the activation rate constant is increased (similar to Fig. S3 in [1]).

**S2 Fig.**  Effects of variations in the value of kcat3.

The total SOS concentration in the upstream GF-SOS model is 50 nM (see Table S1 in S1 Text). Therefore kcat3 value should be high enough for RasGTP to be able to switch between its two steady-states in this range of SOS concentration. As seen in S2 Fig., this is possible for kcat3=1.75 but not for the other values. Therefore we nominally choose kcat3=1.75s^-1^ and K5m=18 nM. The resulting bistable regime is shown in S3 Fig.

**S3 Fig.** The bistable regime for RasGTP after parameter tuning. kcat3=1.75s^-1^ and K5m=18 nM.

1. **Effect of RGAP**

Since increase in the concentration of RGAP increases deactivation, the bistable regime shifts to higher SOS concentrations as shown in S4 Fig. Bistability is robust with respect to this parameter as well.

**S4** **Fig**. Effects of variations in the value of RGAP.

1. **Effect of** $\boldsymbol{\beta}$

The total concentration of Ras has the effect of scaling the response curve as shown in S5 Fig.

**S5 Fig**. Effect of $\beta$ on the bistable regime.

Based on this sensitivity analysis we choose the following values for the tuned parameters:

kcat3=1.75s^-1^ and K5m=18 nM, $\beta=200 (nM)$ and RGAP =0.1 (nM). The rest of the parameters are fixed at their original values [1] given in Table S2 in S2 text.

**Reference:**

[1] J. Das *et al.*, “Digital Signaling and Hysteresis Characterize Ras Activation in Lymphoid Cells,” *Cell*, vol. 136, no. 2, pp. 337–351, 2009.
