# Supplementary material for: Dynamics and control of the ERK signaling pathway: Sensitivity, bistability, and oscillations
Source: PLoS One. 2018 Apr 9;13(4):e0195513. doi: 10.1371/journal.pone.0195513 (PMC5891012; doi:10.1371/journal.pone.0195513)
Supplement: S3 Text — (DOCX) [file pone.0195513.s008.docx]

S3 Text. MAPK subsystem model

This model is borrowed from [1]. The parameters and reactions are listed in Table S3.

Table S3- Parameters and reactions for the MAPK subsystem [1].

| **Parameter values** | **Reactions** |
| --- | --- |
| **a1**=0.0056; **d1**=4.3517; **k1**=2.4345; | 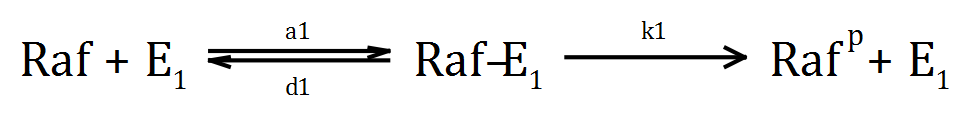 |
| **a2**=0.0307; **d2**=3.3078; **k2**=5.6407; | 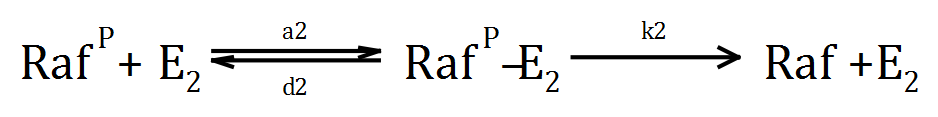 |
| **a3**=0.0204; **d3**=10.3862; **k3**=7.0000; | 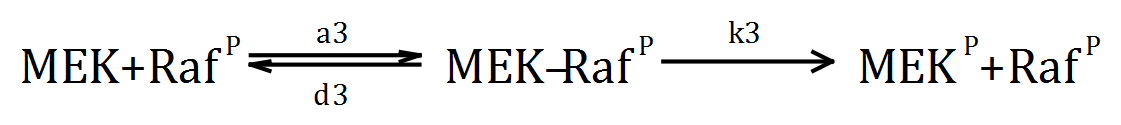 |
| **a4**=0.0493; **d4**=2.7167; **k4**=11.1367; | 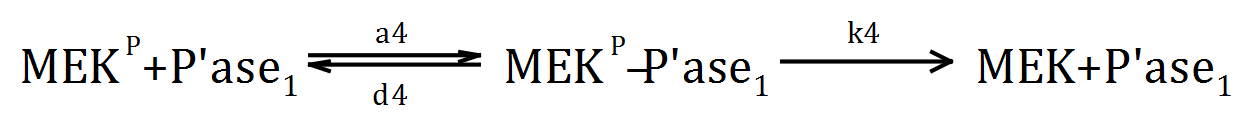 |
| **a5**=0.0564; **d5**=10.0885; **k5**=3.5775; | 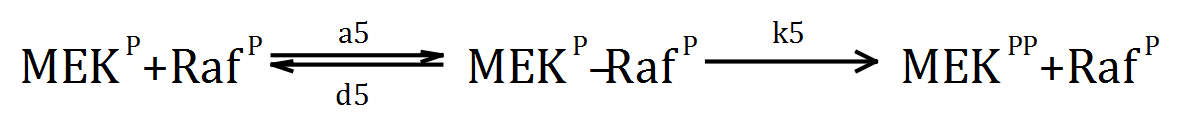 |
| **a6**=0.0326; **d6**=0.8134; **k6**=1.1328; | 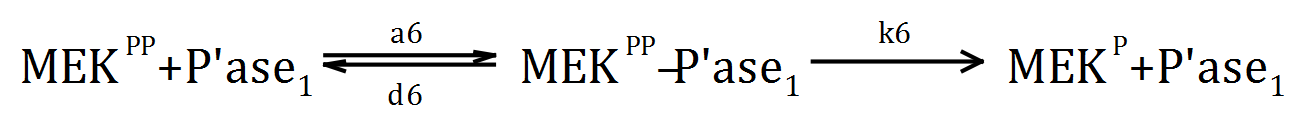 |
| **a7**=0.0038; **d7**=11.5688; **k7**=0.7276; | 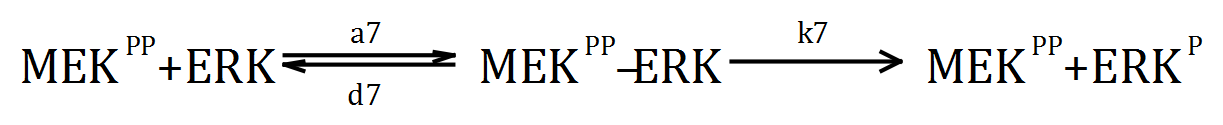 |
| **a8**=0.0050; **d8**=5.0182; **k8**=0.5291 | 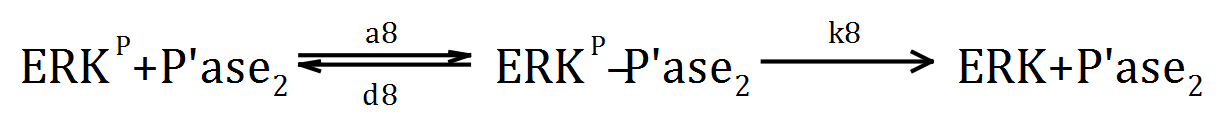 |
| **a9**=0.0056; **d9**=8.0892; **k9**=1.0955 | 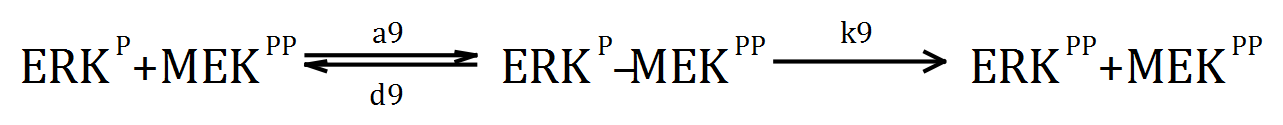 |
| **a10**=0.0162;**d10**=9.7908;**k10**=2.9318 | 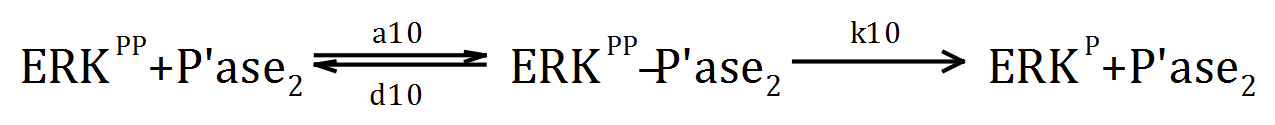 |
| **Conserved moieties (nM)** | |
| [Raf _tot_]= 9.2235e-1, [MEK _tot_]= 5.1288e2, [ERK _tot_]= 8.1552e2, [E_1 tot_]**= 5e-2, [E_2 tot_]= 3.2830e-1,  [P’ase1 _tot_]= 2.1238e-1, [P’ase2 _tot_] =5.0345e2 | |

** [E_1_] is substituted with RasGTP in the complete model.

**Reference:**

[1] L. Qiao, R. B. Nachbar, I. G. Kevrekidis, and S. Y. Shvartsman, “Bistability and oscillations in the Huang-Ferrell model of MAPK signaling,” *PLoS Comput. Biol.*, vol. 3, no. 9, pp. 1819–1826, 2007.
