# Supplementary material for: Dynamics and control of the ERK signaling pathway: Sensitivity, bistability, and oscillations
Source: PLoS One. 2018 Apr 9;13(4):e0195513. doi: 10.1371/journal.pone.0195513 (PMC5891012; doi:10.1371/journal.pone.0195513)
Supplement: S4 Text — (DOCX) [file pone.0195513.s009.docx]

S4 Text- Parameters (estimated) and reactions for the external feedback loops.

| **Parameter values** | **Reactions** |
| --- | --- |
| gpT=10 $(nM.s^{-1})$ |  |
| KmT=1000 $(nM)$ |  |
| dT=1 $(s^{-1})$ |  |
| gpA=1e-6 $(nM.s^{-1})$ |  |
| KmA=10 $(nM)$ |  |
| dA=1e6 $(s^{-1})$ |  |
| kAE=5 $(nM^{-1}.s^{-1})$ |  |
| dAE=1 $(s^{-1})$ |  |
