# Supplementary material for: Dynamics and control of the ERK signaling pathway: Sensitivity, bistability, and oscillations
Source: PLoS One. 2018 Apr 9;13(4):e0195513. doi: 10.1371/journal.pone.0195513 (PMC5891012; doi:10.1371/journal.pone.0195513)
Supplement: S5 Text — (DOCX) [file pone.0195513.s010.docx]

### S5 Text Model equations.

Table A- Model variables vs. molecule names.

| Variable | Molecule Name | Variable | Molecule name |
| --- | --- | --- | --- |
| z1 | EGF_EGFR | z2 | (EGF_EGFR)2 |
| z3 | (EGF_EGFR)2-P | z4 | (EGF_EGFR)2_PLCg |
| z5 | (EGF_EGFR)2_PLCg-P | z6 | (EGF_EGFR)2_Grb2 |
| z7 | (EGF_EGFR)2_Grb2_SOS | z8 | (EGF_EGFR)2_Shc |
| z9 | (EGF_EGFR)2_Shc-P | z10 | (EGF_EGFR)2_Shc_Grb2 |
| z11 | (EGF_EGFR)2_Shc_Grb2_Sos | z12 | Grb2_SOS |
| z13 | Shc-P | z14 | Shc_Grb2 |
| z15 | PLCg-P | z16 | PLCg-P-I |
| z17 | Shc_Grb2_SOS or SOS_complex_ | z18 | EGFR |
| z19 | EGF | z20 | PLCg |
| z21 | Grb2 | z22 | Shc |
| z23 | SOS |  | |
| Rd | RasGDP | Rt | RasGTP |
| SRd | SOS_complex_-RasGDP | SRt | SOS_complex_-RasGTP |
| x1 | Raf_RasGTP |  | |
| x2 | Raf* | x3 | Raf*_E2 |
| x4 | MEK_Raf* | x5 | MEK-P |
| x6 | MEK-P_P'ase 1 | x7 | MEK-P_Raf* |
| x8 | MEK-PP | x9 | MEK-PP_P'ase 1 |
| x10 | ERK_MEK-PP | x11 | ERK-P |
| x12 | ERK-P_P'ase 2 | x13 | ERK-P_MEK-PP] |
| x14 | ERK-PP | x15 | ERK-PP_P'ase 2 |
| x16 | Raf | x17 | MEK |
| x18 | ERK | x19 | - |
| x20 | E2 | x21 | P'ase 1 |
| x22 | P'ase 2 |  | |

Table B- Total amount of conserved moieties (nM).

| EGFR | tot1=10; |
| --- | --- |
| EGF | tot2=0.1; |
| PLCg | tot3=105; |
| Grb2 | tot4=85; |
| Shc | tot5=150; |
| SOS | tot6=50 |
| Ras | tot7=200 |
| GAPS | Rgap=0.1 |
| Raf | tot8=9.2235e-001 |
| MEK | tot9=5.1288e+003 |
| ERK | tot10=8.1552e+002 |
| RasGTP (included in tot7) | tot11=5e-002 |
| E2 | tot12=3.2830e-001 |
| P’ase 1 | tot13=2.1238e-001 |
| P’ase 2 | tot14=5.0345e+002 |

Algebraic equations representing conserved moieties:

| $z18=tot1-\left( z1+2*\left( z2+z3+z4+z5+z6+z7+z8+z9+z10+z11 \right) \right)-grAE*[ARGOS-EGFR]$ |
| --- |
| $z19=tot2+grT*TACE-(z1+2*(z2+z3+z5+z4+z8+z9+z6+z7+z10+z11))$ |
| $z20=tot3-(z4+z5+z15+z16)$ |
| $z21=tot4-(z12+z14+z17+z6+z7+z10+z11+SRd+SRt)$ |
| $z22=tot5-(z13+z14+z17+z8+z9+z10+z11+SRd+SRt)$ |
| $z23=tot6-(z12+z17+z7+z11+SRd+SRt)$ |
| $Rd=tot7-(Rt+SRd+SRt+x1)$ |
| $x16=tot8-(x2+x1+x3+x4+x7)$ |
| $x17=tot9-(x5+x8+x4+x7+x6+x9+x10+x13)$ |
| $x18=tot10-(x11+x14+x10+x13+x12+x15)$ |
| $x20=tot12-(x3)$ |
| $x21=tot13-(x6+x9)$ |
| $x22=tot14-(x12+x15)$ |

GF-SOS complex reactions:

| $r1=kf1*z18*z19-kb1*z1$ |
| --- |
| $r2=kf2*z1*z1-kb2*z2$ |
| $r3=kf3*z2-kb3*z3$ |
| $r4=Vu4*z3/(Ku4+z3)$ |
| $r5=kf5*z3*z20-kb5*z4$ |
| $r6=kf6*z4-kb6*z5$ |
| $r7=kf7*z5-kb7*z3*z15$ |
| $r8=Vu8*z15/(Ku8+z15)$ |
| $r9=kf9*z3*z21-kb9*z6$ |
| $r10=kf10*z6*z23-kb10*z7$ |
| $r11=kf11*z7-kb11*z3*z12$ |
| $r12=kf12*z12-kb12*z21*z23$ |
| $r13=kf13*z3*z22-kb13*z8$ |
| $r14=kf14*z8-kb14*z9$ |
| $r15=kf15*z9-kb15*z13*z3$ |
| $r16=Vu16*z13/(Ku16+z13)$ |
| $r17=kf17*z9*z21-kb17*z10$ |
| $r18=kf18*z10-kb18*z3*z14$ |
| $r19=kf19*z10*z23-kb19*z11$ |
| $r20=kf20*z11-kb20*z17*z3$ |
| $r21=kf21*z13*z21-kb21*z14$ |
| $r22=kf22*z14*z23-kb22*z17$ |
| $r23=kf23*z17-kb23*z13*z12$ |
| $r24=kf24*z9*z12-kb24*z11$ |
| $r25=kf25*z15-kb25*z16$ |

1. **The rate of the species changes (ODEs) for GF-SOS complex subsystem model:**

| $\frac{dz1}{dt}=r1-2*r2$ |
| --- |
| $\frac{dz2}{dt}=r2+r4-r3$ |
| $\frac{dz3}{dt}=r3+r7+r11+r15+r18+r20-r4-r5-r9-r13$ |
| $\frac{dz4}{dt}=r5-r6$ |
| $\frac{dz5}{dt}=r6-r7$ |
| $\frac{dz6}{dt}=r9-r10$ |
| $\frac{dz7}{dt}=r10-r11$ |
| $\frac{dz8}{dt}=r13-r14$ |
| $\frac{dz9}{dt}=r14-r24-r15-r17$ |
| $\frac{dz10}{dt}=r17-r18-r19$ |
| $\frac{dz11}{dt}=r19-r20+r24$ |
| $\frac{dz12}{dt}=r11+r23-r12-r24$ |
| $\frac{dz13}{dt}=r15+r23-r21-r16$ |
| $\frac{dz14}{dt}=r18+r21-r22$ |
| $\frac{dz15}{dt}=r7-r8-r25$ |
| $\frac{dz16}{dt}=r25$ |
| $\frac{dz17}{dt}=-r23+r22+r20-kfi1*z17*Rd+kbi1*SRd-kfi2*z17*Rt+kbi2*SRt-k\_nfb*z17*x14/(km\_nfb+z17)$ |

1. **The rate of the species changes (ODEs) for the Ras subsystem model:**

| $\frac{dSRd}{dt}=kfi1*z17*Rd-kbi1*SRd+kfi2*z17*Rt-kbi2*SRt-(kfi2*z17*Rt-kbi2*SRt)$ |
| --- |
| $\frac{dSRt}{dt}=kfi2*z17*Rt-kbi2*SRt$ |
| $\frac{dRt}{dt}=-kfi2*z17*Rt+kbi2*SRt+(kcati3*Rd*SRt)/(ki3m+Rd)+(kcati4*Rd*SRd)/(ki4m+Rd)-(kcati5*Rgap*Rt)/(ki5m+Rt)-a1*x16*Rt+(d1+k1)*x1$ |

1. **The rate of the species changes (ODEs) for the MAPK subsystem model:**

| $\frac{dx1}{dt}= a1*x16*Rt- (d1+k1)*x1$ |
| --- |
| $\frac{dx2}{dt}= k1*x1- a2*x2*x20+ d2*x3- a3*x2*x17+(k3+d3)*x4- a5*x5*x2+(k5+d5)*x7$ |
| $\frac{dx3}{dt}= a2*x2*x20-(d2+k2)*x3$ |
| $\frac{dx4}{dt}= a3*x17*x2-(d3+k3)*x4$ |
| $\frac{dx5}{dt}= k3*x4-a4*x5*x21+d4*x6-a5*x5*x2+d5*x7+k6*x9$ |
| $\frac{dx6}{dt}= a4*x5*x21-(d4+k4)*x6$ |
| $\frac{dx7}{dt}= a5*x5*x2-(d5+k5)*x7$ |
| $\frac{dx8}{dt}= k5*x7-a6*x8*x21+d6*x9-a7*x8*x18+(d7+k7)*x10-a9*x11*x8+(d9+k9)*x13$ |
| $\frac{dx9}{dt}= a6*x8*x21-(d6+k6)*x9$ |
| $\frac{dx10}{dt}=a7*x18*x8-(d7+k7)*x10$ |
| $\frac{dx11}{dt}=k7*x10-a8*x11*x22+d8*x12-a9*x11*x8+d9*x13+k10*x15$ |
| $\frac{dx12}{dt}=a8*x11*x22-(d8+k8)*x12$ |
| $\frac{dx13}{dt}=a9*x11*x8-(d9+k9)*x13$ |
| $\frac{dx14}{dt}=k9*x13-a10*x14*x22+d10*x15$ |
| $\frac{dx15}{dt}=a10*x14*x22-(d10+k10)*x15$ |

The rate of the species changes (ODEs) for external feedback loops model:

| $\frac{d[TACE]}{dt}=gpT*\frac{x14}{KmT+x14}-dT*[TACE]$ |
| --- |
| $\frac{d[ARGOS]}{dt}=gpA*\frac{x14}{KmA+x14}-dA*[ARGOS]$ |
| $\frac{d[ARGOS-EGFR]}{dt}=kAE*z18*\left[ ARGOS \right]-dAE*[ARGOS-EGFR]$ |
